# Self-sustaining charge circulation in FeS_2_/MoS_2_ heterostructures for micropollutant removal

**DOI:** 10.1016/j.ese.2026.100699

**Published:** 2026-04-13

**Authors:** Zhengyi Lu, Yuxiang Hong, Jiefeng Xiao, Qian Zhang, Han Feng, Junming Hong

**Affiliations:** a College of Chemical Engineering, Huaqiao University, Fujian, Xiamen, 361021, China; b Xiamen Engineering Research Center of Industrial Wastewater Biochemical Treatment, Xiamen, 361021, China; c Fujian Provincial Research Center of Industrial Wastewater Biochemical Treatment (Huaqiao University), Fujian, Xiamen, 361021, China

**Keywords:** Redox shuttle, Charge circulation, Dual active sites, Radical and nonradical synergy

## Abstract

Advanced oxidation processes are widely utilized to eliminate persistent organic pollutants from water. However, their practical effectiveness is significantly constrained by irreversible catalyst deactivation and limited tunability of oxidant pathways. In peroxymonosulfate activation, a central challenge remains sustaining metal redox turnover while maintaining complementary reactive oxygen species under prolonged operation. Here we show that a FeS_2_/MoS_2_ heterointerface functions as an internal redox shuttle, driving a self-sustaining charge-circulation loop that autonomously regenerates dual active sites. A built-in electric field enforces directional electron transfer from Mo to Fe, thereby stabilizing continuous iron redox cycling for the production of radicals (•OH and SO_4_^•−^). Simultaneously, this architecture enables Mo-mediated generation of nonradical singlet oxygen, which mitigates catalyst deactivation and sustains oxidant output. As a result, the system achieves rapid removal of acetaminophen and retains 91.5% of its catalytic activity after 3000 min of continuous operation in various water matrices. These findings establish self-sustaining interfacial charge circulation as a broadly applicable and highly effective strategy for designing robust catalysts for sustainable water treatment.

## Highlights

• A built-in electric field in FeS_2_/MoS_2_ drives a self-sustaining charge circulation loop.
• This internal redox shuttle enables the autonomous real-time regeneration of active sites.
• Dual active sites synergistically drive radical and nonradical pollutant degradation.
• The catalytic system retains 91.5% activity over 3000 min of continuous flow operation.

## Graphical abstract

**Figure:**
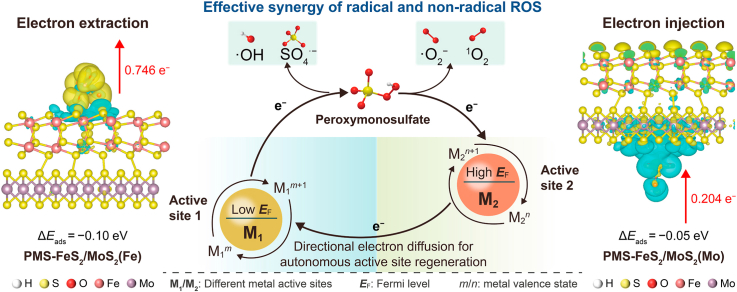

## 1 Introduction

In recent years, increasing concern has been expressed about the presence of pharmaceuticals and personal care products (PPCPs) in natural water bodies [1,2]. These emerging contaminants are highly persistent, poorly biodegradable, and prone to bioaccumulation in aquatic organisms and humans, posing long-term ecological and health risks [3,4]. Among the available options for removing such recalcitrant pollutants, advanced oxidation processes (AOPs) based on Fenton-like reactions have emerged as one of the most effective strategies [[5], [6], [7]]. These systems typically rely on electron transfer at catalytic active sites to generate reactive oxygen species (ROS) and realize organic compound elimination [8], especially in systems combining radical and nonradical pathways, where singlet oxygen has been reported to enable rapid degradation, and free radicals like hydroxyl and sulfate radicals drive deep mineralization [9]. However, the redox cycling of active sites is often hindered by kinetic barriers, leading to irreversible deactivation, diminished catalyst longevity, and impaired process sustainability [10,11]. This not only compromises treatment efficiency but also increases operating costs and environmental burdens, thereby posing significant challenges for practical applications [12].

**Fig. 1:**
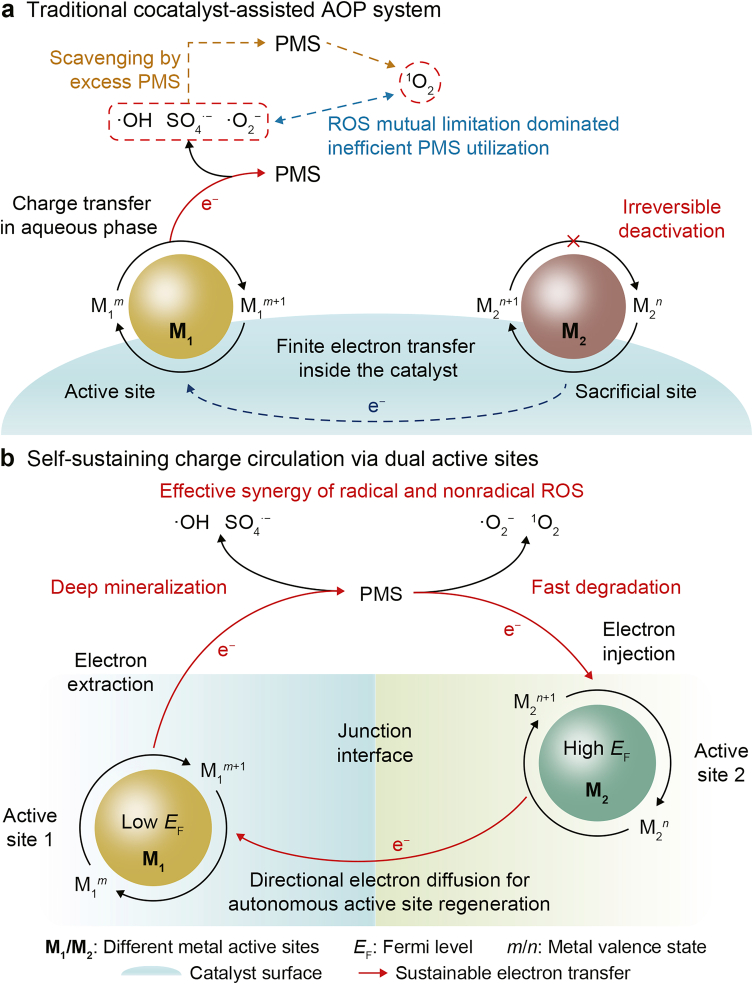
Conceptual comparison of peroxymonosulfate (PMS) activation in traditional and self-sustaining advanced oxidation process systems. **a**, Traditional cocatalyst-assisted system limited by finite electron transfer, reactive oxygen species (ROS) scavenging, and sacrificial-site deactivation. **b**, Dual-active-site system featuring a self-sustaining charge circulation loop for active-site regeneration and synergistic ROS production. AOP, advanced oxidation process; *E*_F_, Fermi level; M_1_/M_2_, different metal active sites; *m*/*n*, metal valence states.

To sustain the activity of catalytic active sites, sacrificial sites are commonly employed to rapidly replenish electrons lost during peroxymonosulfate (PMS) reduction [13]. However, their nonregenerable nature fundamentally limits their ability to maintain long-term active-site functionality. Furthermore, because sacrificial sites do not directly engage with PMS, the derived ROS might be competitively scavenged by excess PMS via side reactions, thereby competing with pollutant oxidation and reducing the effective utilization of both PMS and the radicals (Fig. 1a) [14,15].

To overcome these limitations, we propose a new catalytic architecture incorporating a redox-flexible secondary active site (active site 2) that not only engages in PMS oxidation to generate a broader spectrum of ROS but also donates electrons via a highly efficient and directional electron transfer pathway to regenerate the primary active site (active site 1), while simultaneously undergoing self-regeneration. This dual-active-site system enables a redox-shuttle-mediated charge-circulation pathway that enhances ROS diversity and supports the autonomous regeneration of both active sites, ultimately improving catalytic efficiency and long-term operational stability (Fig. 1b).

In semiconductor physics, when two materials with different work functions form a heterojunction, interfacial carrier redistribution occurs, leading to the formation of a space charge region, band bending, and a built-in electric field (BIEF) [[16], [17], [18], [19]]. While this internal field suppresses the diffusion of majority carriers under thermal equilibrium, thereby preventing further charge redistribution, it can assist or even drive the directional migration of charge carriers under external bias or nonequilibrium injection conditions [20]. Such a mechanism enables efficient charge separation and transport in applications ranging from optoelectronics [21] and electronic devices [22] to catalysis [23,24]. Therefore, we hypothesized that in PMS-involved AOPs, constructing a junction from two materials with distinct work functions and each hosting a reactive site for PMS reduction and oxidation could enable efficient directional charge transfer across the interface, facilitating the autonomous activation of both active sites. This architecture could also establish a dynamic balance of electron extraction and injection during the reaction, thereby enhancing catalyst stability and enriching the diversity of ROS, ultimately leading to significantly improved catalytic performance.

To validate our hypothesis, we selected FeS_2_ and MoS_2_ to construct a heterojunction-based AOP catalyst, leveraging their distinct work functions and complementary abilities in PMS reduction and oxidation. A FeS_2_/MoS_2_ heterostructure was engineered by *in situ* growth of both components on ascorbic acid (AA)-etched Fe_3_O_4_ microparticles, thereby generating abundant heterointerfaces while retaining the magnetic core structure. In this architecture, FeS_2_ provides iron-sulfur redox motifs [[25], [26], [27]], and MoS_2_ provides conductive, edge-rich sulfide layers. Together, they form an efficient electron-transfer platform for PMS activation and offer a model system for investigating self-sustaining interfacial charge circulation, active-site regeneration, and coupled radical/nonradical oxidation pathways.

## 2 Materials and methods

### 2.1 Chemicals and materials

Detailed information on the chemicals and reagents is provided in Supplementary Text S1.

### 2.2 Catalyst preparation

We synthesized FeS_2_/MoS_2_ using a facile one-step hydrothermal method. First, 1.21 g of Na_2_MoO_4_·2H_2_O, 1.52 g of thiourea, 1.18 g of Fe_3_O_4_, and 0.12 g of ascorbic acid were thoroughly mixed with 70 mL of deionized water. The mixtures were then transferred to a 100 mL polytetrafluoroethylene (PTFE)-lined stainless steel hydrothermal vessel, which was placed in an oven at 200 °C for 24 h. The prepared materials were then collected and washed several times with deionized water and ethanol, followed by oven drying overnight at 80 °C. The final products were labeled as FeS_2_/MoS_2_. For comparison, catalysts synthesized without AA (Fe/MoS_2_) or without adding iron precursors (AA-MoS_2_), and catalysts obtained with neither Fe_3_O_4_ nor AA added (MoS_2_) were also prepared under otherwise identical conditions.

### 2.3 Characterization

Material characterizations, including X-ray diffraction (XRD), scanning electron microscopy (SEM), X-ray photoelectron spectroscopy (XPS), and high-resolution transmission electron microscopy (HRTEM), are detailed in Supplementary Text S2. Electrochemical analysis is provided in Supplementary Text S3. PMS consumption, radical quantification, H_2_O_2_ detection, and electron paramagnetic resonance (EPR) analysis are described in Supplementary Texts S4–S7. For details on specific density functional theory (DFT) calculations, refer to Supplementary Text S8. The methods for plant cultivation and toxicity assessment are provided in Supplementary Text S9.

### 2.4 Experimental procedures

Degradation experiments were conducted in 150 mL glass beakers at room temperature. In a typical run, 0.02 g of catalyst (corresponding to 0.2 g L^−1^) and 1 mL of PMS solution (1 mmol L^−1^) were added to 100 mL of acetaminophen (APAP) solution (10 mg L^−1^) to initiate the reaction. At predetermined time intervals, 1 mL of the reaction mixture was withdrawn using a syringe, immediately quenched with 0.5 mL of methanol, and filtered through a 0.22 μm polyethersulfone membrane. The residual concentration of APAP was determined by high-performance liquid chromatography (HPLC, 1260 Infinity Ⅱ, Agilent Technologies, Waldbronn, Baden-Württemberg, Germany) (Supplementary Table S1). All experiments were conducted in triplicate to ensure reproducibility and data reliability.

## 3 Results and discussion

### 3.1 Characterization of materials

**Fig. 2:**
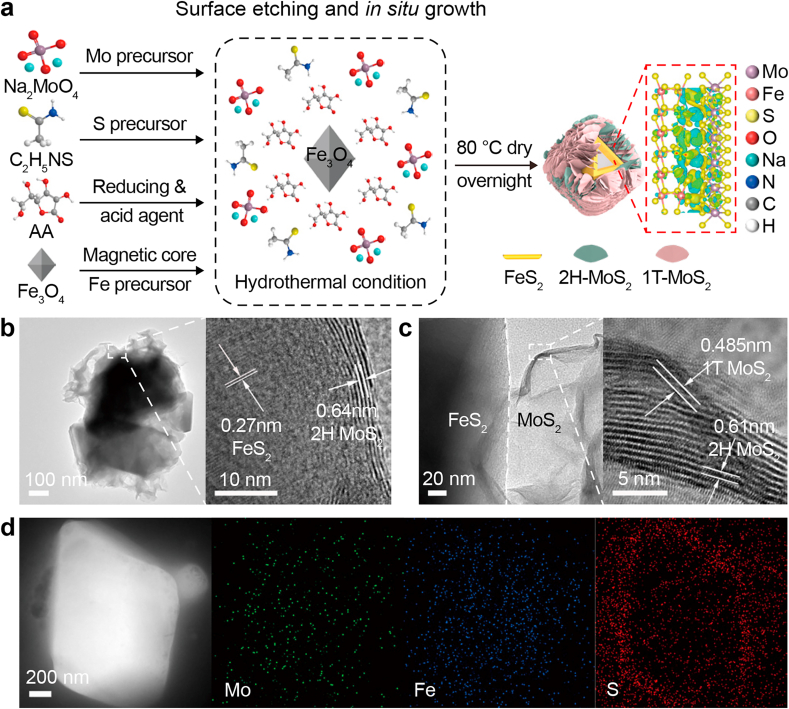
**a**, The schematic illustration of the material preparation procedures. AA, ascorbic acid. **b**–**d**, Morphology characterization of FeS_2_/MoS_2_: transmission electron microscopy (**b**), high-resolution transmission electron microscopy (**c**), and energy-dispersive X-ray spectroscopy elemental mapping images (**d**).

We synthesized the FeS_2_/MoS_2_ heterojunction with a facile one-step hydrothermal method under a reductive atmosphere provided by AA (Fig. 2a). The mildly acidic environment led to the slight etching of Fe_3_O_4_, releasing iron ions that readily reacted with sulfur to form FeS_2_, which subsequently promoted the *in situ* growth of MoS_2_ and the formation of a well-defined FeS_2_/MoS_2_ interface. AA also induced the 2H-to-1T phase transition in MoS_2_, thereby lowering the electron-transfer barrier and reducing the work function [28]. Due to the Fermi level mismatch between FeS_2_ and 1T-MoS_2_, a BIEF formed at the interface, enabling directional electron transfer within the heterostructure [29,30].

Octahedral Fe_3_O_4_ particles (200–1000 nm, Supplementary Fig. S1a) served as both iron sources and a magnetic core for ease of catalyst recovery. The combined presence of AA and Fe_3_O_4_ was essential for the uniform anchoring of MoS_2_ nanosheets on the Fe_3_O_4_ surface (Supplementary Fig. S1b**)**. In contrast, the absence of either component led to non-interfacial MoS_2_ (Supplementary Fig. S1c) or poor nanosheet formation (Supplementary Fig. S1d). Transmission electron microscopy and HRTEM analyses (Fig. 2b and c) revealed a clear core-shell structure with lattice fringes of 0.27 nm and 0.64 nm, corresponding to the (200) plane of FeS_2_ and the (002) plane of MoS_2_, respectively, confirming successful heterojunction formation [13]. High-resolution imaging also identified 1T-MoS_2_ domains, indicative of a lower work function and enhanced electron-donating ability [31], further corroborated by elemental mapping (Fig. 2d).

Next, we examined the crystal phase composition and chemical environment of the FeS_2_/MoS_2_ heterostructure. XRD analysis (Supplementary Fig. S2) revealed the coexistence of characteristic diffraction peaks assigned to Fe_3_O_4_ (JCPDS 15-9961), MoS_2_ (JCPDS 37-1492), and FeS_2_ (JCPDS 42-1340). Raman spectra (Supplementary Fig. S3) exhibited three additional peaks at 147 cm^−1^ (J_1_), 214 cm^−1^ (J_2_), and 336 cm^−1^ (J_3_), along with a characteristic peak at 282 cm^−1^ (E^1^_1g_) for the 2H phase, indicating the coexistence of 1T and 2H polymorphs in MoS_2_ [32].

Fourier transform infrared spectroscopy spectra (Supplementary Fig. S4) showed characteristic peaks at 862 cm^−1^ and 466 cm^−1^ (Supplementary Fig. S4), which are assigned to S–S stretching vibrations and Mo–S or Fe–S bond vibrations, respectively [33], consistent with the formation of a sulfidated surface layer. In addition, the broad absorption band near 3400 cm^−1^, attributed to surface hydroxyl groups, became more pronounced upon AA addition, suggesting enhanced surface hydrophilicity and improved dispersion of the catalyst in aqueous media (Supplementary Fig. S5). FeS_2_/MoS_2_ exhibited the largest specific surface area while maintaining a comparable pore size compared to Fe/MoS_2_ and pristine MoS_2_ (Supplementary Fig. S6), indicating a higher density of accessible active sites and improved adsorption capability, both of which are favorable for enhancing catalytic reactivity [34,35].

**Fig. 3:**
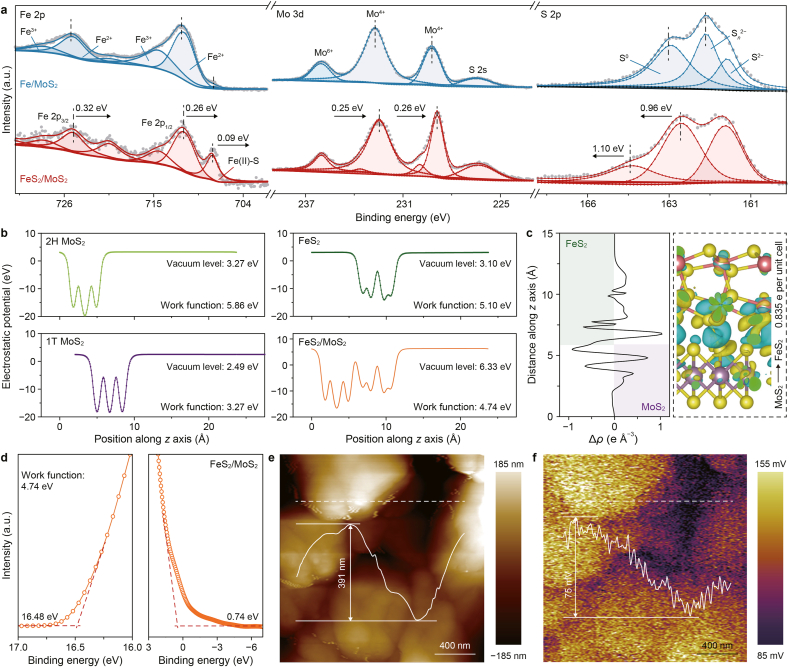
a, X-ray photoelectron spectroscopy spectra of Fe 2p, Mo 3d, and S 2p of as-prepared catalysts. **b**, Plane-averaged electrostatic potential and vacuum level of 2H MoS_2_, 1T MoS_2_, FeS_2_, and FeS_2_/MoS_2_ heterostructure. **c**, Plane-averaged charge density difference (left) and charge redistribution heterointerface (right) for the FeS_2_/MoS_2_ interface. **d**–**f**, Ultraviolet photoelectron spectroscopy spectra (**d**), atomic force microscopy image (**e**), and Kelvin probe force microscopy image (**f**) of the FeS_2_/MoS_2_ heterojunction. Red dashed lines in panel **d** indicate the linear extrapolations used to determine the secondary electron cutoff and valence band onset; white dashed lines in panels **e**–**f** mark the line-scan paths.

XPS characterizations were performed to further probe the interfacial electronic structure and BIEF between FeS_2_ and MoS_2_. The wide-scan spectrum (Supplementary Fig. S7) confirmed the presence of Fe, Mo, S, and O elements in the FeS_2_/MoS_2_ heterostructure. In the high-resolution Fe 2p spectrum (Fig. 3a), peaks at 711.6 and 725.2 eV correspond to Fe^2+^, while those at 714.6 and 728.6 eV are attributed to Fe^3+^ [36]. Moreover, the Fe–S bond at ∼707.8 eV exhibited high intensity compared to Fe/MoS_2_, indicating enhanced FeS_2_ content due to AA-induced surface etching and interfacial coupling between Fe and Mo sulfides [13]. A slight negative shift (∼0.3 eV) in binding energy and an increased Fe^2+^/Fe^3+^ ratio suggest elevated electron density around Fe atoms within the heterojunction. The Mo 3d spectrum also shifted to lower binding energies, attributed to the 2H-to-1T phase transition of MoS_2_, which increased electron availability and reduced the work function, thereby promoting BIEF formation and electron injection from MoS_2_ to FeS_2_ [37]. The S 2p spectrum showed an upshift of ∼1.0 eV in FeS_2_/MoS_2_ compared to its counterpart, due to the metallic 1T phase. No sulfur vacancies were detected by AgNO_3_ complexation (Supplementary Fig. S8) or EPR measurements (Supplementary Fig. S9), further confirming the integrity of the sulfide framework [38].

To elucidate the mechanism of interfacial charge transfer and the corresponding BIEF formation, DFT calculations were performed on the plane-averaged electrostatic potential and the charge density difference (CDD). The pristine FeS_2_ and 1T-MoS_2_ possessed work functions of 5.86 and 3.27 eV, respectively, as illustrated by surface electrostatic potential (Fig. 3b), suggesting a thermodynamically favorable driving force for interfacial electron flow from MoS_2_ to FeS_2_ [39]. Upon heterojunction formation, a notable rearrangement of the vacuum level and work function (4.74 eV) was observed, accompanied by a clear potential step across the interface, indicative of the BIEF in the heterostructure. The plane-averaged CDD and charge transfer isosurface (plotted with cyan and yellow areas representing electron loss and gain in Fig. 3c) further verified a distinct electron depletion in the MoS_2_ region, while electrons accumulated on the FeS_2_ side, yielding a net interfacial electron transfer of 0.835 e per unit cell. This theoretically verified unidirectional electron migration further confirms the formation of a BIEF directed from MoS_2_ to FeS_2_, which is expected to facilitate surface redox reaction kinetics by modulating the adsorption strength of reactive species [40], thereby enabling real-time regeneration of the active sites.

To further validate this self-sustaining circulation in the FeS_2_/MoS_2_ heterojunction we constructed, we conducted ultraviolet photoelectron spectroscopy (UPS) and Kelvin probe force microscopy (KPFM) measurements. The characterized work functions of FeS_2_/MoS_2_ (Fig. 3d), AA-MoS_2_ (Supplementary Fig. S10), and FeS_2_ (Supplementary Fig. S11) were 4.74, 4.65, and 5.32 eV (vs. vacuum), respectively, which are roughly consistent with the results from DFT calculations. Furthermore, the valence band maximum of the FeS_2_/MoS_2_ (0.74 eV) was verified as a downward shift of AA-MoS_2_ (0 eV) and an upshift of FeS_2_ (1.66 eV), directly confirming the formation of a BIEF from MoS_2_ to FeS_2_ in the heterojunction [41].

Subsequently, KPFM was employed to visually confirm the presence of surface electrostatic potential on the heterojunction. As characterized by the atomic force microscopy image (Fig. 3e), the 390 nm thickness of the MoS_2_ layer was roughly consistent with the SEM results (Supplementary Fig. S1b), indicating that the selected region encompasses both the MoS_2_ and FeS_2_ surfaces. Consequently, the contact potential difference observed in the KPFM image (Fig. 3f) reveals a significantly lower surface potential of FeS_2_ than that of MoS_2_, visually displaying the BIEF from MoS_2_ to FeS_2_ in the heterojunction we fabricated. The spontaneous electron diffusion from MoS_2_ to FeS_2_ was expected to effectively enhance the Fe^3+^/Fe^2+^ redox cycling, thereby improving PMS activation and promoting the generation of reactive species.

### 3.2 Catalytic performance

**Fig. 4:**
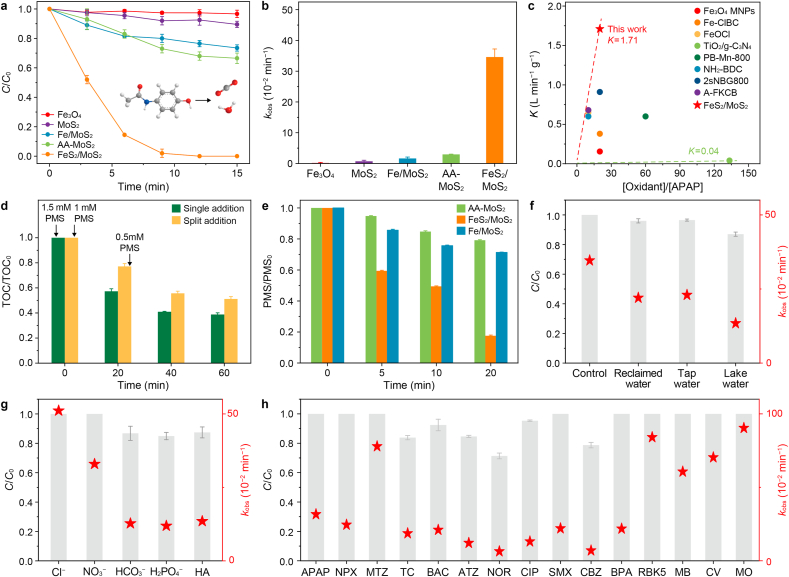
a–**b**, Acetaminophen (APAP) degradation profiles (**a**) and corresponding degradation rate constant (**b**) under various catalytic systems, where AA refers to ascorbic acid. **c**, Comparison of APAP removal efficiency between FeS_2_/MoS_2_ and other reported catalysts. **d**–**e**, Total organic carbon removal (**d**) and peroxymonosulfate (PMS) decomposition (**e**) for different catalytic systems. **f**–**g**, APAP degradation efficiency under complex water matrices (**f**) and various coexisting species (**g**). **h**, Degradation efficiency and rate constants of different pollutants using the FeS_2_/MoS_2_/PMS system, including naproxen (NPX), metronidazole (MTZ), tetracycline (TC), benzalkonium chloride (BAC), atrazine (ATZ), norfloxacin (NOR), ciprofloxacin (CIP), sulfamethoxazole (SMX), carbamazepine (CBZ), bisphenol A (BPA), reactive black 5 (RBK5), methylene blue (MB), crystal violet (CV), and methyl orange (MO). Reaction conditions: catalyst, 0.2 g L^−1^; initial pollutant concentration, 10 mg L^−1^; initial PMS concentration, 1 mmol L^−1^; anions, 10 mmol L^−1^; humic acid (HA), 10 mg L^−1^; reaction time, 15 min; temperature, 25 °C.

The catalytic performance of different catalysts in activating PMS was then systematically evaluated using APAP as the target pollutant, with the optimal catalyst and PMS dosage set at 0.2 g L^−1^ and 1 mmol L^−1^, respectively (Supplementary Fig. S12). All catalysts synthesized showed negligible adsorption (below 10.0%, Supplementary Fig. S13) toward APAP removal before PMS was added. Notably, the FeS_2_/MoS_2_/PMS system achieved nearly complete APAP degradation within only 12 min of adding PMS, outperforming all other systems (Fig. 4a). This enhancement was further supported by pseudo-first-order kinetics, yielding a significantly promoted rate constant (*k*_obs_ = 0.346 min^−1^) (Fig. 4b).

The FeS_2_/MoS_2_/PMS system, with BIEF-induced electron transfer, showed superior catalytic activity (Fig. 4c and Supplementary Table S2), with a catalyst-normalized rate constant (*K*) of 1.71 L min^−1^ g^−1^, outperforming previous systems by 1–2 orders of magnitude. The FeS_2_/MoS_2_/PMS system showed 22.9% mineralization in 20 min with the addition of 1 mmol L^−1^ PMS. When an additional 0.5 mmol L^−1^ PMS was supplemented at 20 min, the mineralization increased to 61.2% at 60 min (Fig. 4d). More than 42.7% mineralization was achieved with a 1.5 mmol L^−1^ PMS dosage at the beginning, which implied the system's robust activation capability toward PMS. The FeS_2_/MoS_2_/PMS system exhibited the highest PMS depletion rate (Fig. 4e) and superior calculated reaction stoichiometric efficiency (Supplementary Fig. S14), highlighting the critical role of BIEF in the heterostructure in enhancing PMS activation and APAP elimination.

The FeS_2_/MoS_2_/PMS system remained highly effective over a wide pH range (3–9) (Supplementary Fig. S15) and superior stability in real water matrices, including lake, tap, and reclaimed water (Fig. 4f). The system retained more than 96.0% APAP removal in tap and reclaimed water, and above 87.0% of the performance was achieved in lake water, despite the relatively high natural organic matter content. The presence of Cl^−^ increased the reaction rate constant from 0.346 to 0.511 min^−1^ due to the formation of reactive chlorine species (HOCl, Cl•, and Cl_2_^•−^) (equations (1), (2), (3), (4)) (Fig. 4g) [42]. Although a slight decrease in activity was observed due to the radical scavenging (equations (5), (6)) in the presence of HCO_3_^−^, H_2_PO_4_^−^, and HA, more than 87.4% of the performance was retained.

(1) Cl^−^ + SO_4_^•−^ → SO_4_^−^ + Cl•

(2) Cl^−^ + HSO_5_^−^ → SO_4_^2−^ + HOCl

(3) Cl^−^ + Cl• →Cl_2_^•−^

(4) Cl• + Cl• →Cl_2_

(5) H_2_PO_4_^−^ + SO_4_^•−^→ SO_4_^2−^ + H_2_PO_4_^•−^

(6) HCO_3_^−^ + SO_4_^•−^→ SO_4_^2−^ + CO_3_^•−^+ H^+^

Apart from APAP, the FeS_2_/MoS_2_/PMS system exhibited remarkable versatility in refractory pollutant degradation (Fig. 4h and Supplementary Table S3), including naproxen (NPX), metronidazole (MTZ), tetracycline (TC), benzalkonium chloride (BAC), atrazine (ATZ), norfloxacin (NOR), ciprofloxacin (CIP), sulfamethoxazole (SMX), carbamazepine (CBZ), bisphenol A (BPA), reactive black 5 (RBK5), methylene blue (MB), crystal violet (CV), and methyl orange (MO). More importantly, the FeS_2_/MoS_2_/PMS system also achieved complete degradation of a mixed-pollutant system containing five representative compounds, including APAP, MTZ, BPA, RBK5, and MB (Supplementary Fig. S16), within 15 min. These results highlight the high-performance applicability and broad adaptability of the Fenton-like system with radical and nonradical synergy.

Magnetic measurements (Supplementary Fig. S17) confirmed the superparamagnetic nature of the FeS_2_/MoS_2_ heterostructure, enabling easy recovery and regeneration through external magnetic fields. These findings confirm that the FeS_2_/MoS_2_ catalyst exhibits outstanding stability and generalizability, both of which are critical for effective catalyst recovery and regeneration.

### 3.3 Long-term stability and electron transfer pathway

**Fig. 5:**
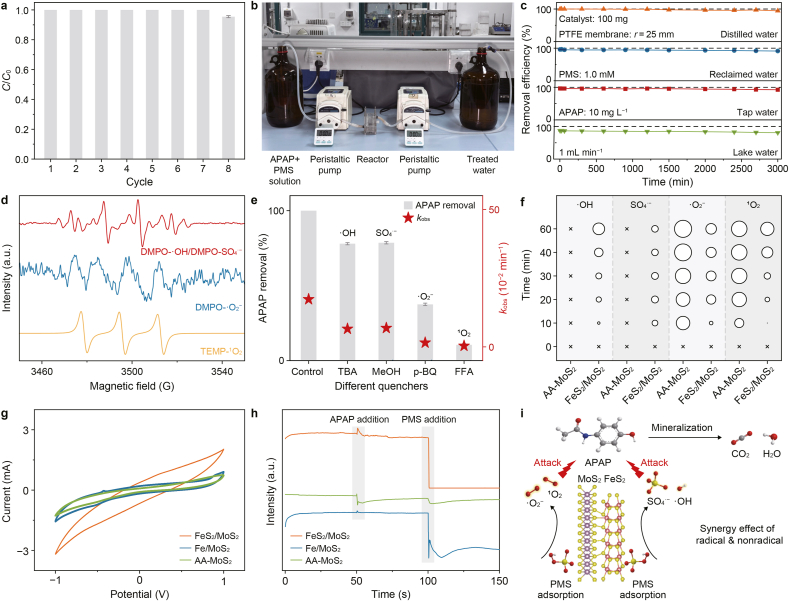
a, Acetaminophen (APAP) degradation efficiency under batch cycling experiments. **b**, Photograph of the continuous-flow reactor used for APAP/PMS treatment, where PMS denotes peroxymonosulfate. **c**, Time-resolved APAP removal performance in different water matrices using the continuous-flow reactor equipped with a PTFE (polytetrafluoroethylene) membrane. **d**, Electron paramagnetic resonance spectra of DMPO-•OH/DMPO- SO_4_^•−^, DMPO-•O_2_^−^, and TEMP-^1^O_2_ in the FeS_2_/MoS_2_/PMS system. **e**, APAP removal efficiency and corresponding degradation rate constant under different quenching conditions; the symbols above the bars indicate the reactive oxygen species probed by the corresponding quenchers. **f**, •O_2_^−^, ^1^O_2_, •OH, and SO_4_^•−^ quantification of different catalysts. × indicates an undetectable or negligible signal; circles indicate detectable reactive oxygen species; circle size is proportional to the relative signal intensity; AA, ascorbic acid. **g**–**h**, Cyclic voltammograms (**g**) and time-dependent current responses to sequential APAP and PMS addition (**h**) for FeS_2_/MoS_2_, Fe/MoS_2_, and AA-MoS_2_. **i**, Radical generation route in FeS_2_/MoS_2_/PMS system. Reaction conditions: initial APAP concentration, 10 mg L^−1^; catalyst, 0.2 g L^−1^; initial PMS concentration, 1 mmol L^−1^; methanol (MeOH), 100 mmol L^−1^; tert-butanol (TBA), 100 mmol L^−1^; p-benzoquinone (p-BQ), 10 mmol L^−1^; furfuryl alcohol (FFA), 10 mmol L^−1^; reaction time, 15 min; initial pH, 7.

The real-time regeneration of active reaction sites could, in theory, substantially enhance the catalyst's sustainability and long-term stability. To verify this, we first conducted batch experiments, and more than 95.5% of its original performance was retained even after eight consecutive cycles (Fig. 5a), demonstrating the impressive recyclability of this system. The resulting SO_4_^2−^ level (Supplementary Fig. S18) was below the Chinese Surface Water Environmental Quality Standard (GB 3838-2002, [SO_4_^2−^] ≤ 250 mg L^−1^).

To verify the catalyst's practical adaptability, long-term tests were conducted using a continuous-flow reactor setup (Fig. 5b). The system achieved over 91.5% APAP removal efficiency in distilled, reclaimed, and tap water, and maintained a respectable performance even in lake water after 3000 min of uninterrupted operation (Fig. 5c). Post-reaction XRD patterns (Supplementary Fig. S19) confirmed that the FeS_2_/MoS_2_ structure retained its crystalline structure intact. Substantially reduced Fe and Mo ion leaching (82.4% and 93.1% reductions, respectively) compared to Fe/MoS_2_ further underscores the advantage of BIEF-mediated real-time active site regeneration, which significantly enhances the catalysts' stability and sustainability.

To examine the ROS involved in PMS activation, we conducted EPR spectroscopy and radical-quenching experiments. EPR spectra confirmed the presence of multiple ROS (Fig. 5d), including SO_4_^•−^, •OH, •O_2_^−^, and ^1^O_2_ in the FeS_2_/MoS_2_/PMS system [43]. Radical-scavenging experiments further clarified their contributions to APAP degradation [44] (Fig. 5e and Supplementary Fig. S20). Notably, ^1^O_2_ exhibited a highly efficient and rapid degradation capacity toward the target pollutant [45,46], while •O_2_^−^, as the key precursor of ^1^O_2_, played a crucial role in sustaining its generation. The superior APAP removal in D_2_O compared to H_2_O (Supplementary Fig. S21) further supports ^1^O_2_ involvement due to its extended lifetime in deuterated solvent. In contrast, •OH and SO_4_^•−^ contributed less to the rapid removal of the pollutant, as their primary function lies in the deeper mineralization of intermediate products. These findings underscore the advantage of the synergistic mechanism, both radical and nonradical, in achieving both efficient degradation and complete water purification.

Next, dissolved oxygen (DO) and H_2_O_2_ were investigated to verify the origin of ROS [47]. N_2_-purging significantly suppressed APAP degradation (*k*_obs_ from 0.35 min^−1^ to 0.19 min^−1^, Supplementary Fig. S22), confirming the participation of DO. H_2_O_2_ generation was detected via colorimetric methods (Supplementary Fig. S23–S25), and both Fe/MoS_2_/PMS and FeS_2_/MoS_2_/PMS systems showed clear H_2_O_2_ signals. However, when H_2_O_2_ was used as a substitute oxidant, no characteristic 652 nm absorption of TMB-•O_2_^−^ complex was observed (Supplementary Fig. S26), suggesting that the •O_2_^−^ detected in PMS systems was not derived from H_2_O_2_ intermediates. Instead, H_2_O_2_ likely decomposed into more reactive •OH and ^1^O_2_ species.

To further delineate the respective roles of FeS_2_ and MoS_2_ sites in PMS activation, ROS quantification experiments were conducted across different systems (Fig. 5f and Supplementary Material Fig. S27–S29). Notably, •OH (Supplementary Fig. S29a) and SO_4_^•−^ (Supplementary Fig. S29b) radicals were detected only in Fe/MoS_2_/PMS and FeS_2_/MoS_2_/PMS systems, while remaining almost undetectable in Fe-exclusive systems like MoS_2_/PMS and AA-MoS_2_/PMS systems, indicating that Fe serves as the active site responsible for the generation of these radicals.

Substantial •O_2_^−^ (Supplementary Fig. S29c) and ^1^O_2_ (Supplementary Fig. S29d) were observed in all Mo-involved systems, indicating the significant role of Mo in their generation. Significantly intensified signals were captured in the AA-MoS_2_/PMS system, attributed to the presence of the 1T phase and the increased density of active Mo^4+^ sites introduced by AA modification, which facilitates PMS activation via a nonradical pathway [48]. Thus, in the FeS_2_/MoS_2_/PMS system, Fe sites primarily govern the radical generation through PMS reduction, while Mo sites facilitate the formation of nonradical ^1^O_2_ and •O_2_^−^ via PMS oxidation in comparable amounts. This coexistence of both radical and nonradical ROS, originating from these dual-active-site heterostructures, enables rapid degradation and deep mineralization of APAP molecules, thereby ensuring their complete removal.

To further elucidate electron-transfer behavior in the FeS_2_/MoS_2_/PMS system, we employed electrochemical techniques. FeS_2_/MoS_2_ heterostructures exhibited the largest enclosed area in the cyclic voltammetry test, indicating enhanced electron storage and transfer ability (Fig. 5g). Electrochemical impedance spectroscopy (Supplementary Fig. S30) demonstrated lower charge-transfer resistance, implying enhanced interfacial electron mobility within the FeS_2_/MoS_2_ system. Negligible variation in the current density was recorded upon the addition of APAP (10 mg L^−1^). In contrast, a sharp decrease in the current density was observed upon injection of PMS (10 mmol L^−1^), confirming a pronounced electron redistribution driven by the spontaneous interactions of PMS at the dual active sites (Fig. 5h).

Linear sweep voltammetry curves (Supplementary Fig. S31) captured with and without PMS in both anodic and cathodic scans revealed PMS-involved reduction and oxidation processes on the FeS_2_/MoS_2_ electrode. This indicates that PMS molecules donate electrons at Mo sites to generate ^1^O_2_ and •O_2_^−^, and accept electrons at Fe sites to produce SO_4_^•−^ and •OH. The concurrent generation of radical and nonradical ROS facilitates a cooperative degradation pathway, leading to the complete removal and mineralization of APAP (Fig. 5i). The reduced Mo and oxidized Fe were further recovered through spontaneous electron transfer induced by the BIEF inside the heterostructure, thus forming two redox shuttles and realizing the autonomous regeneration of the active sites.

PMS serves simultaneously as an electron acceptor and donor at Fe and Mo sites, maintaining an internal electron balance at the catalyst surface. This self-sustaining redox behavior prevents deactivation of active sites due to excessive electron depletion, thereby maintaining catalytic efficiency and stability.

### 3.4 Redox shuttle-mediated self-sustaining charge circulation

**Fig. 6:**
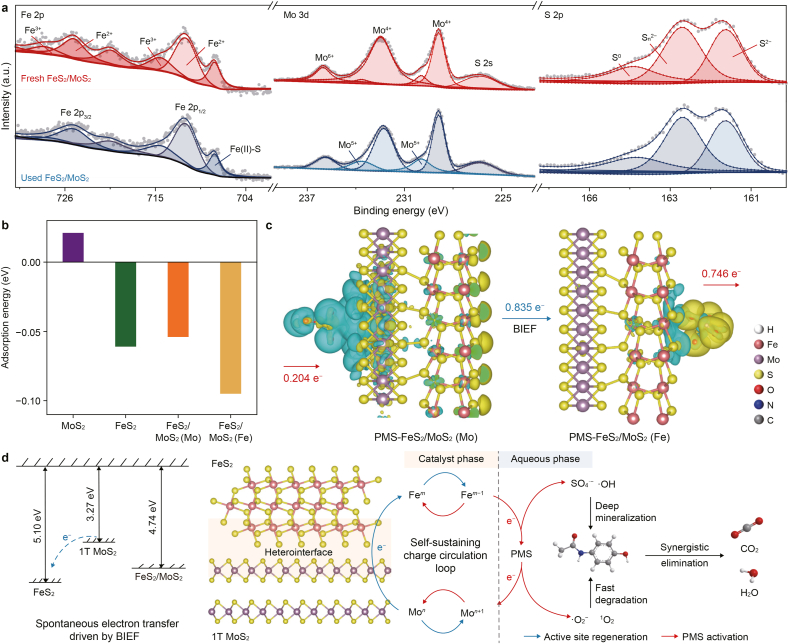
a, X-ray photoelectron spectroscopy spectra of S 2p, Mo 3d, Fe 2p of fresh and used FeS_2_/MoS_2_. **b**, Adsorption energy of peroxymonosulfate (PMS) on different catalyst surfaces. **c**, Electron density difference plot at PMS-FeS_2_/MoS_2_ interface, showing the built-in electric field (BIEF). **d**, Proposed mechanism for the APAP oxidative catalysis in the FeS_2_/MoS_2_/PMS system.

To gain insight into the PMS activation strategy on the surface of the dual-active site heterostructures, XPS analyses were conducted on the fresh and used catalysts (Fig. 6a). The Fe^2+^ content remained nearly unchanged after the reaction, indicating that the Fe active sites were preserved and regenerated during the process, since Fe^2^^+^ ⇌ Fe^3+^ cycling plays a key role in PMS activation. Concurrently, a slight decrease in Mo^4+^ content (15.0%) and an increase in Mo^5+^ (14.9%) were observed, suggesting a slight oxidation of Mo, possibly due to minor nonstoichiometry during interaction with PMS. The content of the active high-valent Mo^6+^ remained nearly unchanged (Supplementary Table S4), likely due to the limited oxidizability of Mo(V) [49]. This indicated the presence of the Mo^6+^ ⇌ Mo^5+^ ⇌ Mo^4+^ redox shuttle, which contributes to the formation of a continuous electron flow within the catalytic system in collaboration with Fe^2^^+^ ⇌ Fe^3+^.

The constant ratio of S_n_^2−^/S^2−^ implies their role as electron reservoirs: S^2−^ is oxidized to S^0^ to donate electrons (S^2−^ → S^0^ + 2e^−^), and S^0^ is subsequently reduced back to S^2−^ by PMS or H_2_O, sustaining the redox cycle [38]. This sulfur-mediated cycle aligns with the BIEF-driven electron transfer from MoS_2_ to FeS_2_, thereby enabling the continuous regeneration of active metal centers with minimal variation in electron density after catalysis.

Next, DFT calculations were performed to validate the redox shuttle-mediated self-sustained regeneration of active sites induced by PMS molecules. Compared with pristine MoS_2_ (0.02 eV) and FeS_2_ (−0.06 eV) (Supplementary Fig. S32), PMS exhibited markedly enhanced adsorption at the heterointerface, with adsorption energies of −0.05 eV at the Mo site and −0.10 eV at the Fe site of FeS_2_/MoS_2_ heterostructures, highlighting strengthened PMS–catalyst interactions and more pronounced interfacial electron redistribution (Fig. 6b). Bader charge analysis further revealed that PMS adsorption on the Fe site led to a significant electron donation of 0.746 e from the catalyst to PMS, whereas PMS adsorption on the Mo site involved a 0.204 e back-transfer to the catalyst (Fig. 6c). Together, the DFT results substantiate the pivotal role of PMS in driving directional electron transfer and inducing the redox shuttle (Fe^2+^ ⇌ Fe^3+^; Mo^5+,6+^ ⇌ Mo^4+^) mediated charge circulation within the heterostructure-mediated catalytic cycle.

Integrating experimental results and theoretical insights, a mechanistic model for the FeS_2_/MoS_2_/PMS/APAP system is proposed (Fig. 6d). First, the BIEF at the FeS_2_/MoS_2_ heterointerface, arising from the lower work function of 1T MoS_2_, drives spontaneous electron migration from MoS_2_ to FeS_2_, thereby generating abundant low-valent Fe sites and high-valent Mo sites. Next, the surface-exposed low-valent Fe sites activate PMS via electron donation to produce SO_4_^•−^ and •OH radicals [50], while the high-valent Mo sites act as electron acceptors to facilitate nonradical PMS activation (Supplementary Fig. S33). This activation process transiently elevates Fe valence and reduces Mo valence, but these active states are rapidly regenerated by the BIEF. Thus, PMS functions as an electron shuttle in the aqueous phase, establishing a self-sustained charge circulation loop within the FeS_2_/MoS_2_/PMS system. Under the continuous drive of this loop, radical and nonradical reactive species are persistently generated at Fe and Mo sites, respectively, enabling fast degradation and deep mineralization of APAP. As a result, APAP was completely degraded within 12 min, with a mineralization rate of 42.7% within 20 min, while retaining near-unity catalytic activity after eight consecutive cycles.

### 3.5 Reactive site identification and degradation pathway of APAP

**Fig. 7:**
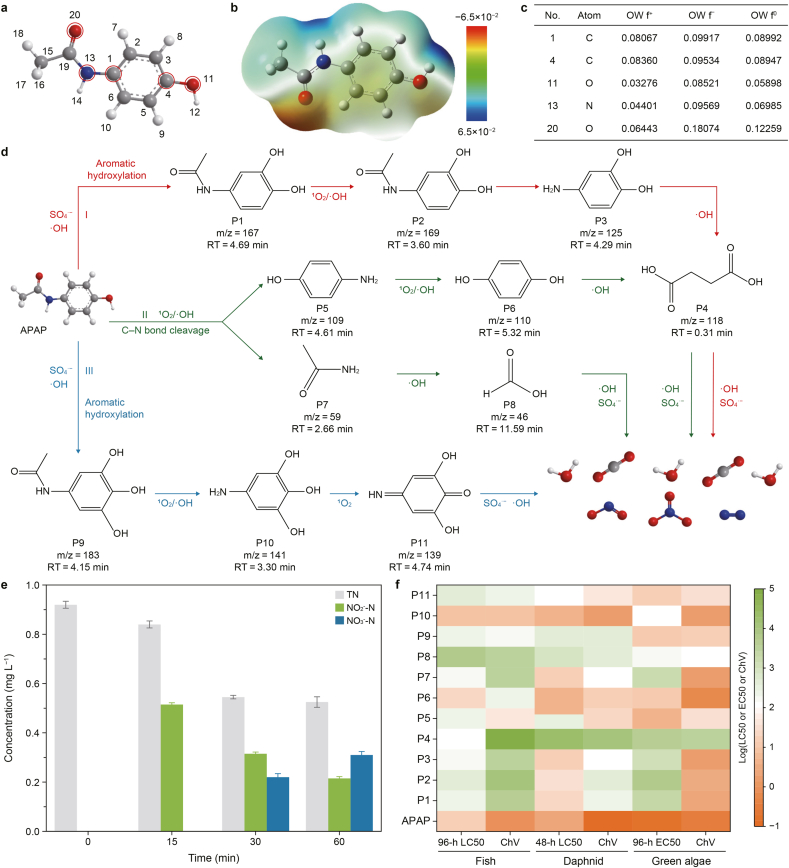
a–**b**, Chemical structure (**a**) and electrostatic potential distribution (**b**) of acetaminophen (APAP). Circles in panel **a** highlight the atoms discussed in the Fukui function analysis in panel **c**. **c**, Fukui function values of the highlighted atoms. “No.” denotes the atom numbering corresponding to panel **a**. **d**, Possible pathways for removing APAP. P1–P11 denote the identified transformation products, *m*/*z* refers to the mass-to-charge ratio, and RT refers to the retention time. **e**, Nitrogen species transformation during APAP degradation. TN, total nitrogen. **f**, Estimated log_10_ values of acute and chronic toxicity of APAP and transformation products by ecological structure activity. LC50, median lethal concentration; EC50, median effective concentration; ChV, chronic value.

Based on the chemical structure of APAP (Fig. 7a), electrostatic potential (ESP) (Fig. 7b) and Fukui function analysis (Fig. 7c and Supplementary Table S5) were performed. The results revealed that atoms including 1C, 4C, 11O, 13N, and 20O exhibited relatively high f^−^-values, indicative of their susceptibility to electrophilic attack by singlet oxygen [51]. Among them, 20O showed the highest value, indicating it was the most reactive site. The 1C and 4C sites also displayed elevated values, suggesting their involvement in oxidative reactions. ESP mapping revealed that the electron-rich acetylamino group in APAP served as a preferential site for electrophilic attack by ^1^O_2_ [52,53].

In pathways I and III of aromatic hydroxylation, •OH and SO_4_^•−^ first abstract an electron from the para carbon (4C) of the aromatic ring, generating an unstable APAP radical cation (APAP•^+^) [54] and yielding hydroxylated products P1 and P9 [15] (Fig. 7d and Supplementary Fig. S34). Subsequent oxidation by •OH or ^1^O_2_ converts these intermediates into phenolic compounds (P2–P3 and P10), which are then dehydrogenated by the attack of •OH and SO_4_^•−^ to form P4 and P11. In pathway II (C–N bond cleavage), the acetylamino group in APAP exhibits strong electron-donating characteristics, making it highly susceptible to electrophilic attack by ^1^O_2_ and •OH [55]. Notably, the reactive sites at 1C (f^−^ = 0.099) and 13N (f^−^ = 0.096) are preferentially targeted, leading to cleavage of the C–N bond to form intermediates, including P5 and P7 [56]. Finally, ring-opening reactions are triggered by attack from •OH and SO_4_^•−^, ultimately leading to the formation of inorganic end-products (CO_2_, H_2_O, and N-containing inorganic species).

DFT calculations were performed to obtain the energy profiles of the compounds involved in APAP degradation (Supplementary Table S6), and the reaction-free energies of the three reaction pathways **(**Supplementary Fig. S35) were calculated. The results indicated that all three pathways were thermodynamically feasible, with reaction-free energies of −1.77, −1.92, and −4.52 eV for pathways I, II, and III, respectively. The agreement between the computed intermediates and those detected by LC-MS further validates the plausibility of the proposed degradation mechanisms.

To clarify the final fate of the nitrogen atom, the total nitrogen (TN) and inorganic nitrogen species (NO_2_^−^ and NO_3_^−^) were monitored (Fig. 7e). The results revealed that NO_2_^−^ was detected at the early stage (15 min) and then gradually converted to NO_3_^−^, with 33.7% (ΔNO_3_^−^/TN_0_) of initial total nitrogen being converted to NO_3_^−^, indicating a stepwise oxidation process in which NO_3_^−^ was the dominant terminal nitrogen species. The continuous decline in TN (from 0.92 to 0.53 mg L^−1^) suggests that a portion of nitrogen was removed from the aqueous phase, likely via denitrification, resulting in the formation of gaseous products such as N_2_. Thus, the progressive oxidation of all identified intermediates reflects a well-coordinated, synergistic mechanism involving radicals and nonradicals, resulting in rapid degradation and deep mineralization of APAP in the FeS_2_/MoS_2_/PMS system.

Ecological structure activity relationship predictions for the 11 degradation intermediates indicated significantly lower LC50, EC50, and chronic values (ChV) for most byproducts compared to APAP, suggesting that the degradation products are less toxic and more environmentally friendly (Fig. 7f and Supplementary Table S7). This conclusion was further supported by the hydroponic cultivation of cauliflower seedlings with APAP-contaminated and treated water (Supplementary Fig. S36 and Text S9). Seedlings irrigated with APAP-contaminated water exhibited reduced germination rates and smaller sizes, whereas those grown with treated water exhibited improved growth and higher germination rates, confirming the effective removal of APAP and reduced toxicity of the treated effluent. These findings underscore the environmental suitability and effectiveness of the proposed strategy.

## 4 Conclusion

In this work, we constructed a redox shuttle-mediated, self-regenerating catalytic system by integrating FeS_2_/MoS_2_ heterojunctions with dual active sites and employing PMS as an external charge carrier. The AA-induced formation of mixed-phase 1T/2H MoS_2_ and the spontaneous Fe–Mo interfacial electron transfer established a built-in field that continuously drives redox cycling, enabling the synchronized production of radical (•OH and SO_4_^•−^) and nonradical (^1^O_2_) species at spatially distinct sites for the rapid degradation and deep mineralization of micropollutants. Electrochemical and theoretical analyses confirmed PMS's dual role as both an oxidant and an electron shuttle, effectively preventing active-site deactivation and ensuring long-term operational stability. While the present system has realized a PMS-mediated, self-sustaining charge–circulation loop, it is not yet perfectly stoichiometric and thus remains open to further optimization in future work. The proposed design strategy offers new insights into interface-engineered AOP catalysts, demonstrating broad applicability across complex water matrices and laying the foundation for the future development of high-efficiency, recyclable treatment platforms.

## CRediT authorship contribution statement

**Zhengyi Lu:** Writing – original draft, Visualization, Investigation. **Yuxiang Hong:** Software. **Jiefeng Xiao:** Formal analysis. **Qian Zhang:** Formal analysis. **Han Feng:** Writing – review & editing, Supervision, Formal analysis, Conceptualization. **Junming Hong:** Supervision, Resources, Funding acquisition.

## Declaration of competing interest

The authors declare that they have no known competing financial interests or personal relationships that could have appeared to influence the work reported in this paper.
