# Supplementary material for: Self-sustaining charge circulation in FeS2/MoS2 heterostructures for micropollutant removal
Source: Environ Sci Ecotechnol. 2026 Apr 13;31:100699. doi: 10.1016/j.ese.2026.100699 (PMC13122226; doi:10.1016/j.ese.2026.100699)
Supplement: Multimedia component 1 [file mmc1.docx]

**Supplementary Materials**

**Self-sustaining charge circulation in FeS_2_/MoS_2_ heterostructures for micropollutant removal**

Zhengyi Lu^a,b,c^, Yuxiang Hong^a,b,c^, Jiefeng Xiao^a,b,c^, Qian Zhang^a,b,c^, Han Feng^a,b,c,*^, Junming Hong^a,b,c,*^

^a^ College of Chemical Engineering, Huaqiao University, Fujian, Xiamen 361021, China

^b^ Xiamen Engineering Research Center of Industrial Wastewater Biochemical Treatment, Xiamen 361021, China

^c^ Fujian Provincial Research Center of Industrial Wastewater Biochemical Treatment (Huaqiao University), Fujian, Xiamen 361021, China

Summary Information

Number of Pages: 48 (including cover page)

Figures: S1-S36

Tables: S1-S7

Texts: S1-S9

**Text S1 Reagents and Materials**

Anhydrous methanol (MeOH, 99.9%), anhydrous ethanol (EtOH, 99.7%), ascorbic acid (AA, 99.7%), KCl (99.9%), KHCO_3_ (99.5%), KNO_3_ (99%), H_2_SO_4_ (95%), HNO_3_ (65%), HCl (37%), NaOH (96%), CN_2_H_4_S (99%), Fe_3_O_4_ (98%), and Na_2_MoO_4_⋅2H_2_O (99%) were purchased from Sinopharm Chemical Reagent Co., Ltd. FeS_2_ (99.9% metals basis), Peroxymonosulfate (PMS, KHSO_5_ • 0.5KHSO_4_ • 0.5K_2_SO_4_, 4.5% (active oxygen)), *p*-benzoquinone (P-BQ, 99%), tert-butyl alcohol (TBA, 99%), humic acid (HA, 90%), furfuryl alcohol (FFA, ≥98%), 5,5-dimethyl-1-pyrroline N-oxide (DMPO, 99%), 2,2,6,6-tetramethyl-4-piperidone (TEMP, 99%) were purchased from Shanghai Aladdin Biochemical Technology Co., Ltd. Deionized (DI) water was used in all experiments.

**Text S2 Material Characterizations**

The crystallization was determined by using X-ray diffraction (XRD) patterns recorded with a Bruker D8-advance X-ray powder diffractometer operated at 40 kV voltage and 20 mA current with CuKα radiation (λ=1.5406 Å). The catalysts of particle size and morphology were analyzed by scanning electron microscope (SEM) carried out on a JEOL JEM-7610M equipped with energy dispersive X-ray spectrometer (EDS) analyzed at 15 kV. X-ray photoelectron spectroscopy (XPS) analysis was performed on scanning X-ray microprobe (Kratos Axis Ultra DLD) operated at 15 kV, 1486.71 eV with monochromated Al Kα radiation. High-resolution transmission electronic microscope (HRTEM) and energy dispersive spectroscopy (EDS) elemental mapping spectra were obtained by a JEOL JEM-2100F operating at 200 kV. The high-angle annular dark-field scanning TEM (HAADF-STEM) was recorded by a JEOL JEM-ARM200F operating at 200 kV. Surface functional groups were detected by a Fourier transform infrared spectrometer (FT-IR, Thermo Scientific Nicolet iS5) using KBr as a reference sample. Raman spectra were detected using a Jobin Yvon HR 800 micro-Raman spectrometer at 520 nm. Inductively coupled plasma mass spectroscopy (ICP-MS, Agilent 7700X) was used to determine the contents of Fe and Mo.

**Text S3** **Electrochemical analysis method**

All the electrochemical measurements were conducted at room temperature in a standard three-electrode electrochemical cell with an Ag/AgCl (4 M KCl) reference electrode, a platinum sheet counter electrode and a catalyst-modified glassy carbon working electrode, and the electrolyte was a mixture of 0.1 M Na_2_SO_4_. Homogeneous catalyst ink was first prepared by sonication of 20 mg catalyst powder, 0.1 mL Nafion solution (5 wt%, Sigma-Aldrich) and 1 mL absolute ethanol. Then, 5 μL of the as-prepared catalyst ink was pipetted onto the surface of the glassy carbon electrode, leading to a catalyst loading of ~0.45 mg cm^-2^. The catalyst layer was dried in ambient air before use. All electrochemical data were collected on the CHI 660e electrochemical workstation (Shanghai Chenhua Instrument Co., China). The electrolyte is 0.1 M Na_2_SO_4_ or a mixed solution of 0.1 M Na_2_SO_4_ and 1 mM PMS. The i-t curve was obtained at -0.40 V vs. Ag/AgCl using 0.1 M Na_2_SO_4_ as electrolyte. Electrochemical impedance spectroscopy (EIS) was recorded at -0.4 V vs. Ag/AgCl in the frequency range 10^5^ to 10^-1^ Hz using an AC voltage of 5 mV amplitude and an electrolyte of 0.1 M Na_2_SO_4_.

**Text S4.** **PMS consumption experimental method.**

The quantification of residual PMS was carried out. A 0.5 mL of reaction solution was mixed with 9.5 mL of deionized water, 1 mL of KI (15 mM + 0.42 g HCO_3_^-^), and the mixed solution was shaken for 5 min to ensure the complete reaction between I^-^ and HSO_5_^-^ to produce I_3_^-^ (Eqs. S1-S2). The I_3_^-^ concentration, proportional to PMS, was determined at λ_max_ = 352 nm by UV-vis spectrophotometer.

$\text{2}\text{I}^{\text{-}}\text{ + HS}\text{O}_{\text{5}}^{\text{-}}\text{ + 2}\text{H}^{\text{+}}\text{= HS}\text{O}_{\text{4}}^{\text{·-}}\text{ +}\text{I}_{\text{2}}\text{ +}\text{H}_{\text{2}}\text{O}$ (S1)

$\text{I}^{\text{-}}\text{ + }\text{I}_{\text{2}}\text{= }\text{I}_{\text{3}}^{\text{-}}$ (S2)

The reaction stoichiometric efficiency (RSE) was employed as a signal balancing PMS depletion and mineralization, which could be calculated as follows:

$\text{RSE(TOC)=ΔTOC/ΔPMS}$ (S3)

**Text S5. Quantification of sulfate radicals, hydroxyl radicals, superoxide radicals, monoclinic oxygen and hydrogen peroxide**

1) Quantification of H_2_O_2_ was done by mixing 1 ml of sample (filtered through a 0.45 μm filter) with 1 ml of 3 mol/L sulfuric acid, 1 ml of 0.05 mol/L titanium (IV) oxide bis(oxalate) acid potassium salt hydrate. After color development reaction of 15 min, the mixed solution was analyzed by UV-vis with detection wavelength of 400 nm. The curve plot is shown in Fig. S24.

2) p-Hydroxybenzoic acid (HBA) was used as a probe compound to indicate the generation of sulfate radicals. Quantification of SO_4_^·-^ was carried out by recording the peak area of p-Benzoquinone (P-BQ) in HPLC at 244 nm. (Mobile phase: deionized water, acetonitrile (v/v: 50%/50%); flow rate of 0.8 ml/min).

SO_4_^·-^ can react with HBA to generate BQ (Eq. S4), in which the amount of BQ is proportional to the SO_4_^·-^concentration. The curve plot is shown in Fig. S27.

$\text{HBA}\left( \text{excess} \right)\text{ + S}\text{O}_{\text{4}}^{\text{·-}}\text{ → hydroquinone + PMS}\left( \text{excess} \right)\text{ →BQ}$ (S4)

3) Sodium benzoate (BA) is used as a probe compound to indicate the generation of hydroxyl groups. Under conventional conditions, ·OH was quantified by withdrawing 1 mL of the reaction solution through a 0.22 μm filter tip and adding it to a detection vial, and by recording the peak area of para hydroxybenzoic acid (HBA) generated by the reaction in HPLC at a wavelength of 255 nm (mobile phase: 0.1% trifluoroacetic acid aqueous solution, acetonitrile (v/v: 65%/35%); flow rate of 1 ml/min).

According to the literature the ·OH concentration is 5.87 times the measured concentration of HBA. The curve plot is shown in Fig. S28.

4) Nitroblue tetrazolium (NBT) (25 μmol/L, λ = 259 nm) was used as a probe to quantify superoxide radicals (·O_2_^-^). 2.5 mL of reaction solution was withdrawn into a cuvette and the concentration of NBT was detected by UV-Vis spectrophotometer to quantify ·O_2_^-^ production.

The ·O_2_^-^ produced forms a 1:1 depletion relationship with the NBT^2+^. Therefore, by calculating the loss of the NBT^2+^ (λ = 259 nm), the amount of produced ·O_2_^-^ will be possible to calculate.

$\text{NBT}^{\text{2+}}\text{+}\text{O}_{\text{2}}^{\text{·-}}\text{→}\text{NBT}^{\text{+}}\text{+}\text{O}_{\text{2}}$ (S5)

5) 1,3-Diphenylisobenzofuran (DPBF) (0.1 mmol/L, λ = 410 nm) was used as a trapping agent for the quantification of single-linear oxygen (^1^O_2_). At first, the reaction solution was prepared by dissolving DPBF into ethanol. Then, PMS and catalyst were added to trigger the generation of ^1^O_2_ and other ROSs. While, other ROSs was kindly quenched with the presence of ethanol. After reaction of 5 min, 2.5 mL of the solution from the reaction was withdrawn and filtered through a 0.22 μm filter tip into a cuvette, and the concentration of DPBF was measured by UV-Vis. The concentration of ^1^O_2_ was obtained from the consumed DPBF as their stoichiometric ratio was 1:1.

**Text S6. Method for peroxidase identification in catalyst/H_2_O_2_ systems**

8 μL of 20 mM TMB was added to 170 μL of acetate buffer (0.2 M, pH=3.6). Then 2 μL of 100 mM H_2_O_2_ and 20 μL of 1 mg L^-1^ catalyst were added to the above solution, and the color development was carried out for 3 min. The enzyme activity was determined by the absorbance at 652 nm in UV-vis.

**Text S7 Determination of free radicals and non-radicals**

The reactive free radicals and non-radicals generated in the system were detected by EPR instrument (Bruker EMXPLUS, Germany). The corresponding parameters are as follows: center field 3502.00 G, sweep width 100.0 G, microwave power 6.325 mW, PowerAtten 15.0 dB, microwave frequency 9.82 GHz, sweep time of 30.00 s, modulation amplitude of 1.000 G, modulation frequency 100.00 kHz.Two spin traps were employed to capture free radicals and non-radicals, 5,5-dimethyl-1-pyrroline N-oxide (DMPO > 99%, Aladdin) and 2,2,6,6-tetramethyl-4-piperidinol (TEMP > 99%, Aladdin), respectively. That is, DMPO and TEMP were dissolved in phosphate buffers of pH = 7.4, respectively, resulting in final concentrations of 80 mM and 50 mM. Then, 1 mL sample was withdrawn from the catalyst suspension reaction system (catalyst dosage: 0.2 g L^-1^, APAP concentration: 10 mg L^-1^, and PMS: 0.1 mM) and was filtered using 0.22 µm filter. DMPO or TEMP solution (40 µL) was mixed with filtered sample (40 µL). Pipette the DMPO or TMP mixed solution with a quartz capillary to detect the corresponding signal of the spin trap adduct in the EPR.

**Text S8 Theoretical Calculations**

In our calculations, the initial reactive site of electrophilic APAP is calculated by Gaussian 09. Geometry optimizations were performed with B3LYP functional and a basis set of 6-311g** for all atoms until the structure was free of imaginary frequencies. Then the Multiwfn software was used to analyze and calculate grid data of Fukui function and dual descriptor.

$$f^{\left( 2 \right)}\left( r \right)={[\frac{\partial\eta}{\partial\upsilon\left( r \right)}]}_{N}={[\frac{\partial f\left( r \right)}{\partial N}]}_{\upsilon(r)}$$

where η and N is the chemical hardness and the number of electrons in the system, and υ(r) is the external potential.

$$f^{\left( 2 \right)}\left( r \right)=f^{+}\left( r \right)-f^{-}\left( r \right)={[\rho}_{N+1}\left( r \right)-\rho_{N}\left( r \right)]-{[\rho}_{N}\left( r \right)-\rho_{N-1}\left( r \right)]\approx\rho^{\mathrm{LUMO}}\left( r \right)-\rho^{\mathrm{HOMO}}(r)$$

In this context, the density of an electron is denoted by 𝜌, and the functions $f^{+}\left( r \right)\mathrm{and}f^{-}\left( r \right)$ are defined as Fukui functions. As stated in the original study, the dual descriptor $f^{\left( 2 \right)}\left( r \right)$ will exhibit positive or negative values depending on whether it corresponds to an electrophilic or nucleophilic site. A site with a more negative value is considered to be more susceptible to attack by electrophilic reagents.

The initial structure of MoS_2_ and FeS_2_ was obtained from the Materials Project. The structure optimization of confined (200) crystal plane of FeS_2_, and (002) crystal plane of MoS_2_, with a 15 Å vacuum layer, were conducted by density functional theory (DFT) in the Born-Oppenheimer framework with the CP2K package[1,2], in which a 4 × 5 supercell for FeS_2_ and a 5 × 5 supercell for MoS_2_ were adopted to ensure the lattice mismatch was below 5%. Moreover, the Perdew-Burke-Ernzerhof (PBE)[3] function within generalized gradient approximation (GGA) was carried out using the exchange-correlation function. The mixed-representation basis sets consisted of plane waves with an energy cutoff of 520 Ry and a double-ζ quality local basis set with a single set of polarization functions (DZVP-MOLOPT-SR-GTH) [4]. The 2s and 2p electrons of O atoms, the 3*s*, and 3*p* electrons of S atoms, the 1s electron of H atoms, the 4p, 4d and 5s for Mo atoms and the 3s, 3p, 3d, and 4s electrons for Fe atoms were explicitly treated as valence electrons. Only the Γ-point was used to sample the Brillouin zone as the computational unit cells are adequately large (see below). In addition, all atomic positions were fully relaxed, based on the limited memory BFGS method, until maximum geometry change, root mean square (RMS) geometry change, maximum force, and RMS force reached a tolerance of 3×10^-3^ eV/Å, 1.5×10^-3^ eV/Å, 4.5×10^-4^ eV/Å, and 3×10^-4^ eV/Å respectively.

The dispersion-corrected DFT-D3 method was used to consider the long-range interactions[5]. The adsorption energy (E_ads_) of a formed complex from metal active sites of catalysts and PMS can be calculated using Eq. (1):

E_ads_ = E_complex_ - (E_catalyst_ + E_PMS_) (1)

E_complex_ is the total energy of the catalyst and PMS molecular complex, while E_catalyst_ and E_PMS_ are the total energies of the isolated catalyst and PMS molecules, respectively.

**Text S9 Methods of plants cultures and toxicity test**

Cauliflower was cultured in 3×4 seedling trays using gray-yellow sandy soil from the coastal area of Hui'an County. Four seeds were randomly planted in each compartment, and two experimental groups were prepared. One group was irrigated with the APAP solution before degradation, and the other group with the APAP supernatant after degradation. The irrigation was carried out over a 1-day period using a drip irrigation method, with 5 mL applied to each compartment. The trays were placed under natural indoor lighting conditions for cultivation. The growth of the cauliflower was monitored, including any individual mortality, and photographic records were made. After two weeks, the seedlings were harvested, and the survival rate and growth status were recorded and analyzed.


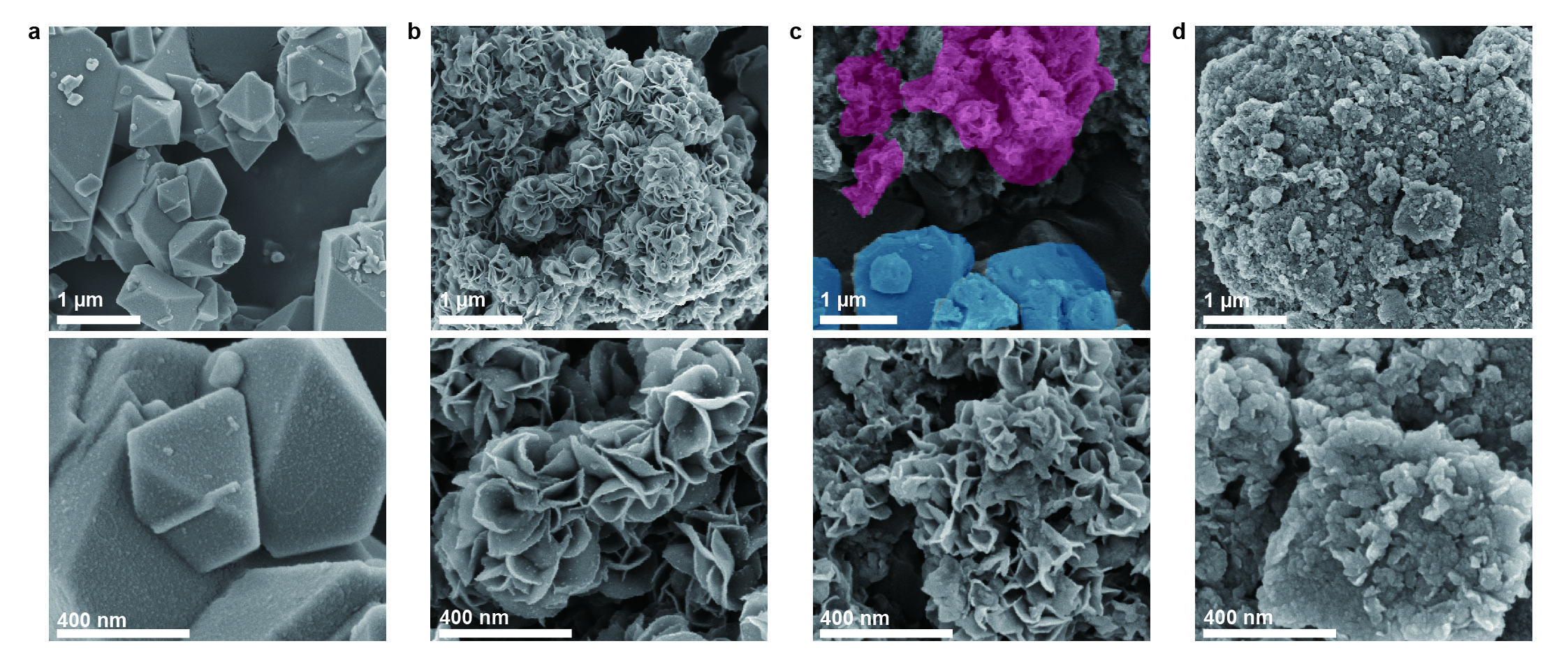


**Fig. S1.** **a-d**, SEM image of Fe_3_O_4_ (**a**)_,_ FeS_2_/MoS_2_ (**b**), Fe/MoS_2_ (**c**), AA-MoS_2_ (**d**).


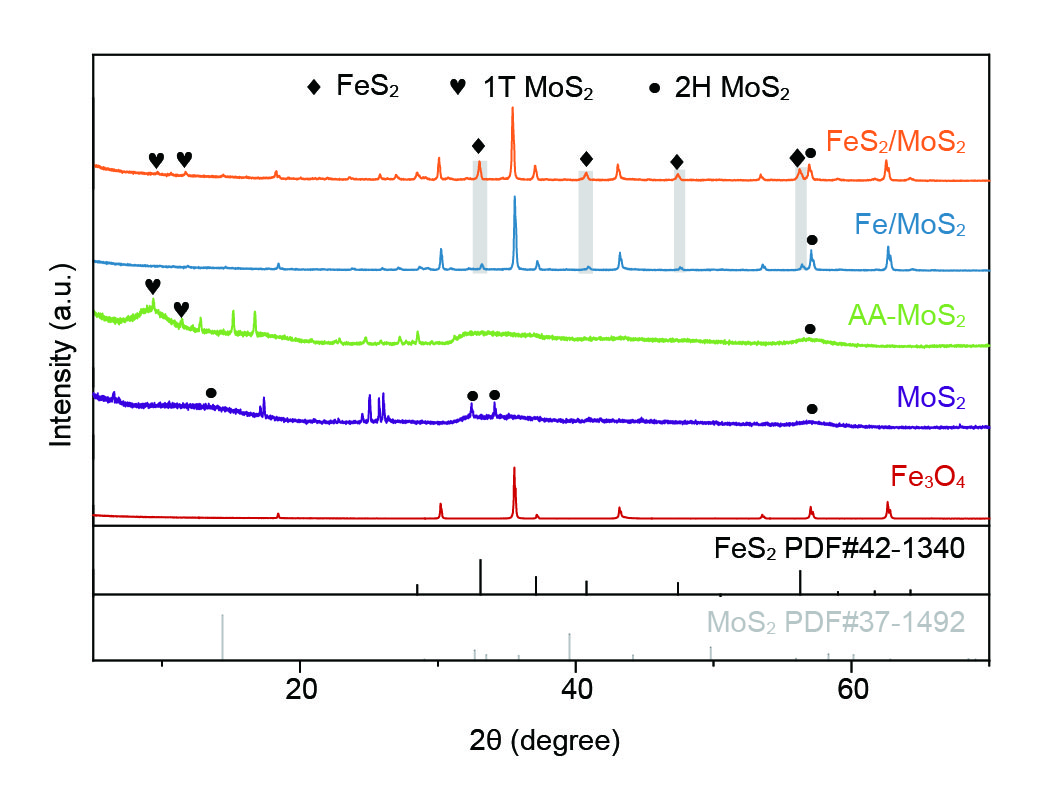


**Fig. S2.** XRD patterns of Fe_3_O_4_, MoS_2_, AA-MoS_2_, Fe/MoS_2_, and FeS_2_/MoS_2_.


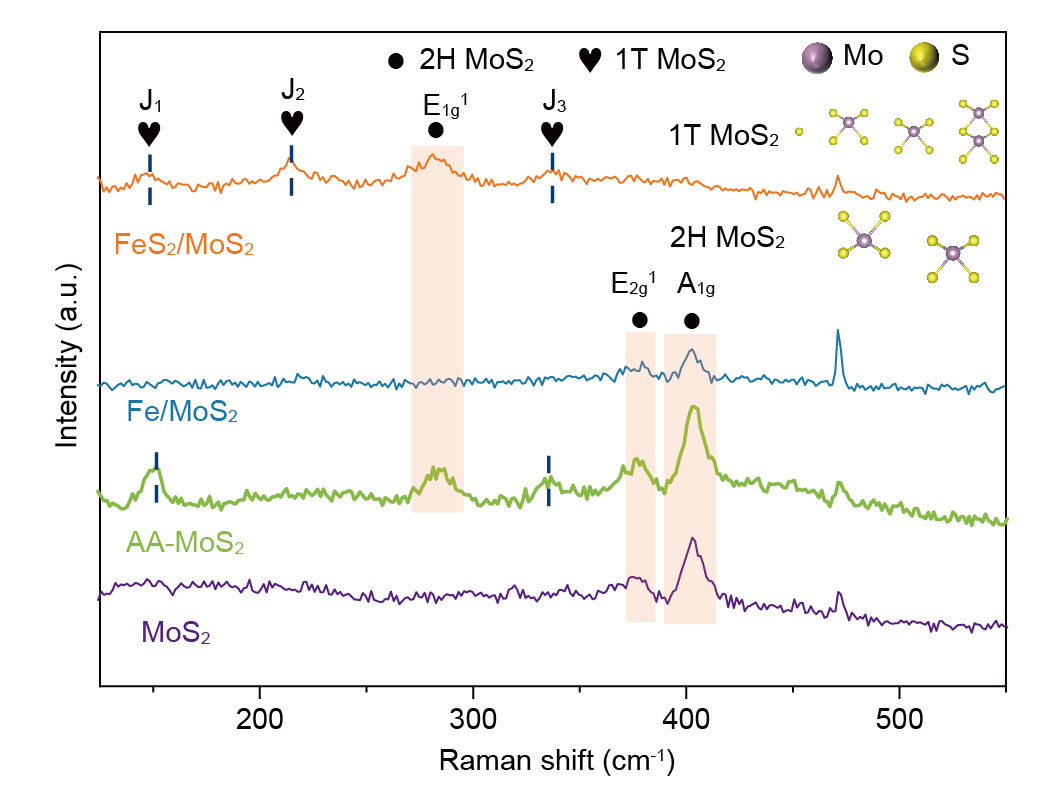


**Fig. S3.** Raman spectra of MoS_2_, AA-MoS_2_, Fe/MoS_2_, and FeS_2_/MoS_2_.

**
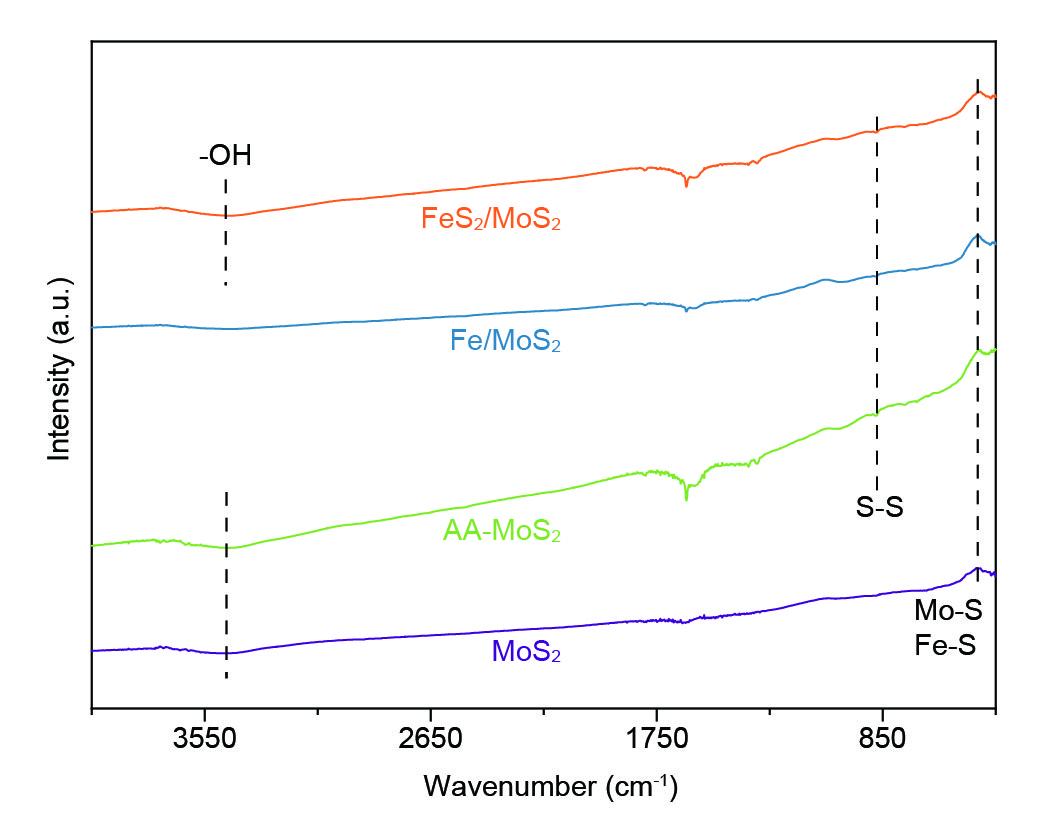
**

**Fig. S4.** FT-IR spectra of MoS_2_, AA-MoS_2_, Fe/MoS_2_, and FeS_2_/MoS_2_.


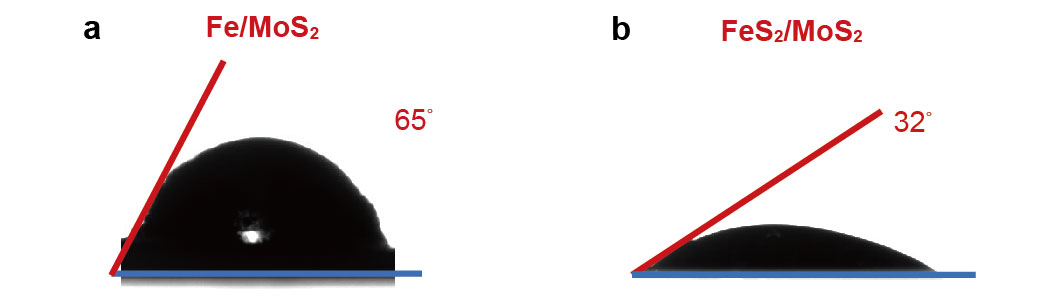


**Fig. S5.** Comparison of water contact angles on Fe/MoS_2_ (**a**) and FeS_2_/MoS_2_ (**b**).


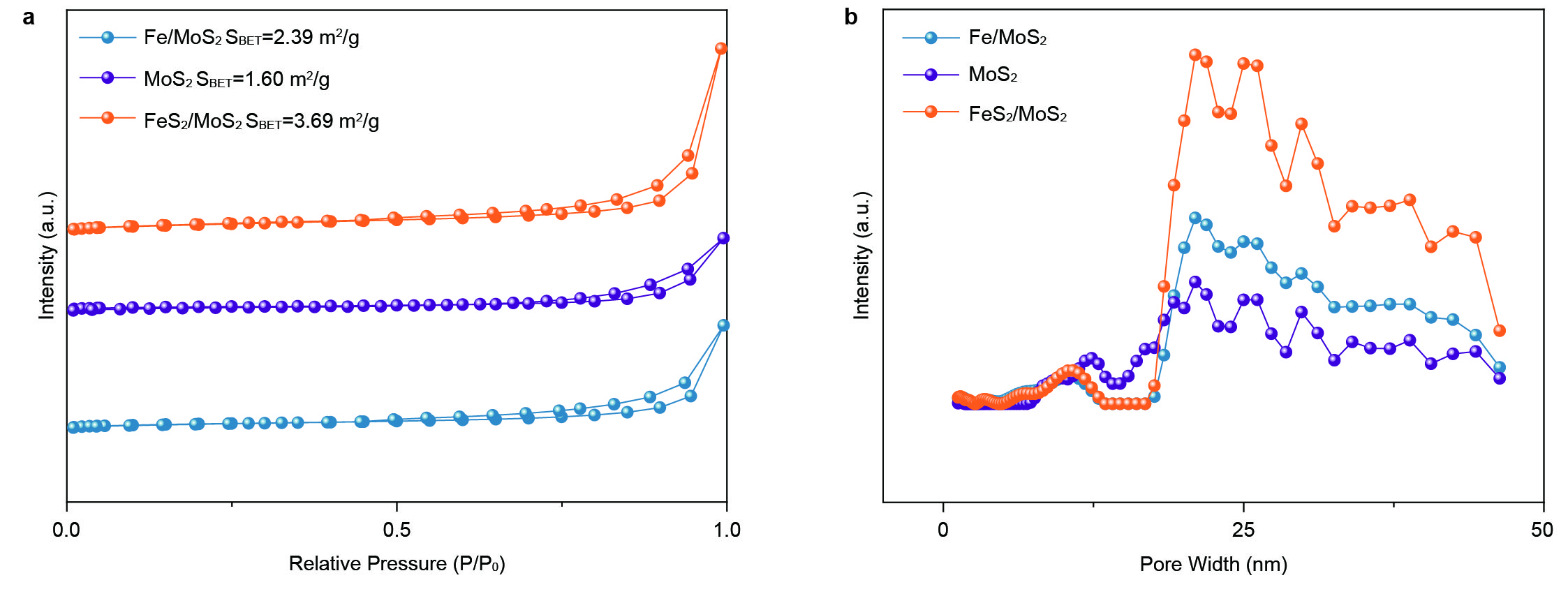


**Fig. S6.** **a-b**, BET measurements (**a**) and corresponding pore size distribution (**b**) of the samples.


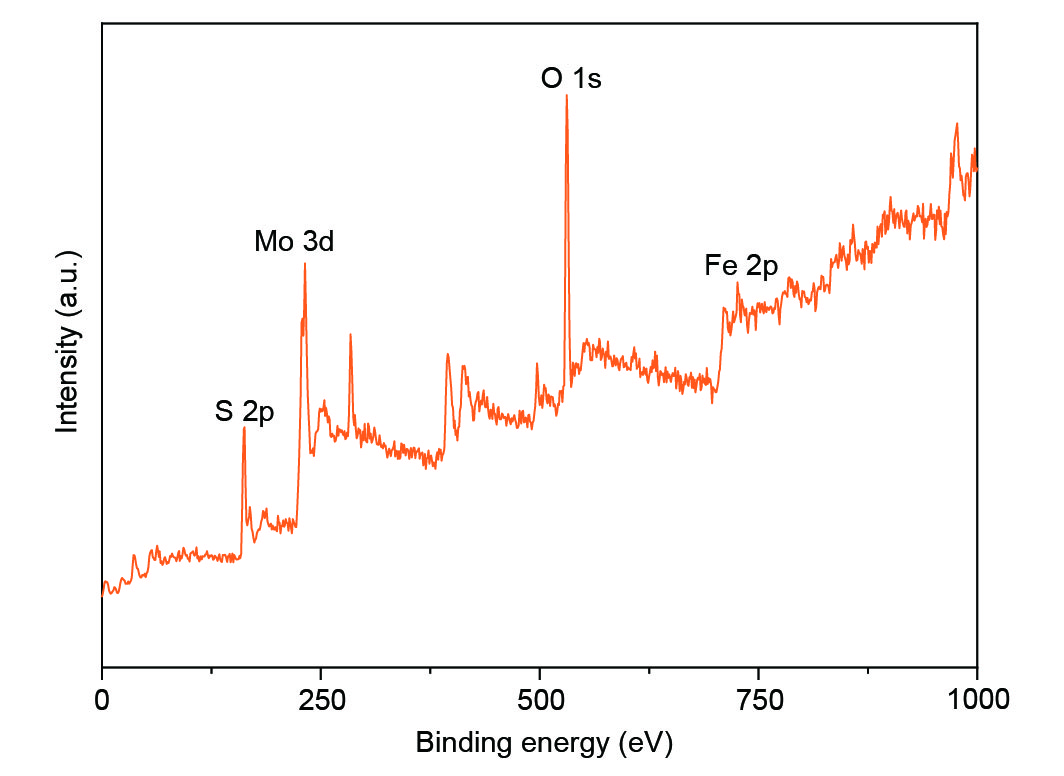


**Fig. S7.** XPS Survey of FeS_2_/MoS_2_.


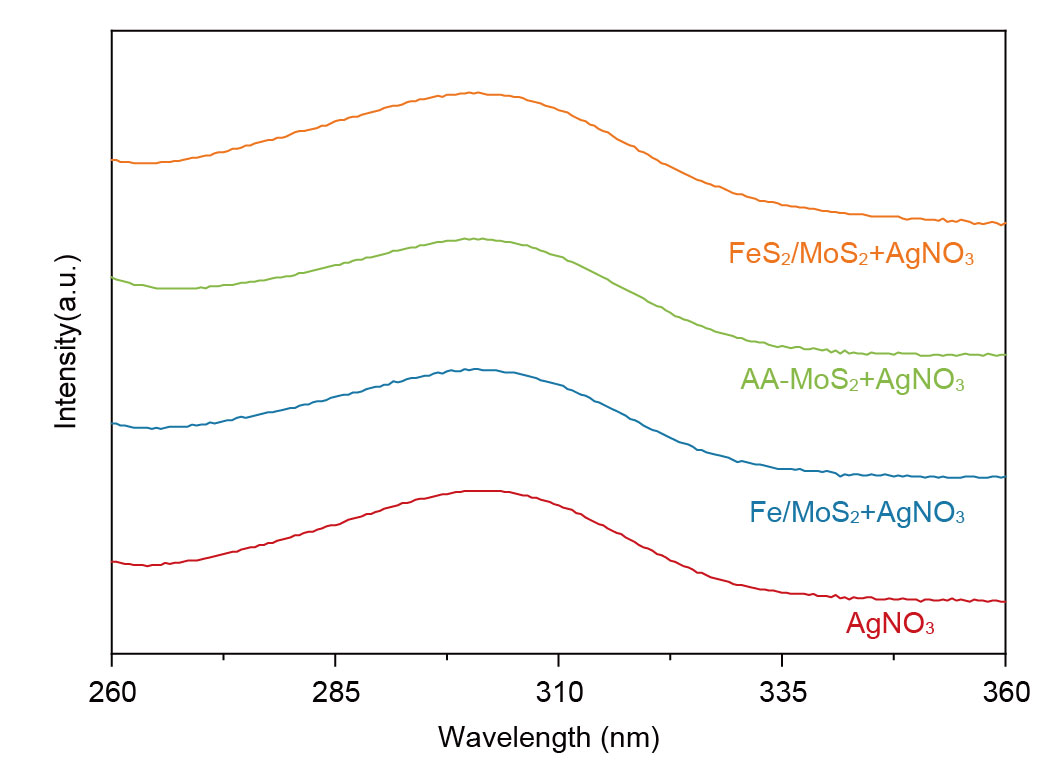


**Fig. S8.** Generation of H_2_S caught by AgNO_3_ during the reactions using different catalysts.


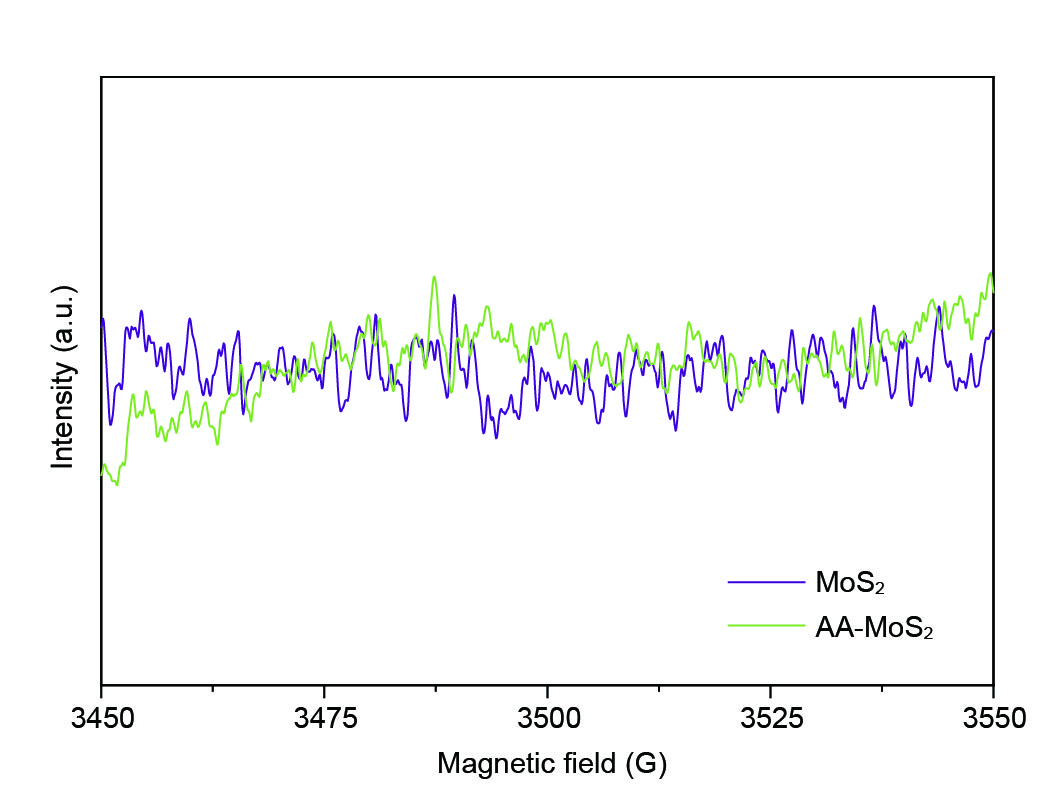


**Fig. S9.** EPR spectra of MoS_2_ and AA-MoS_2_.


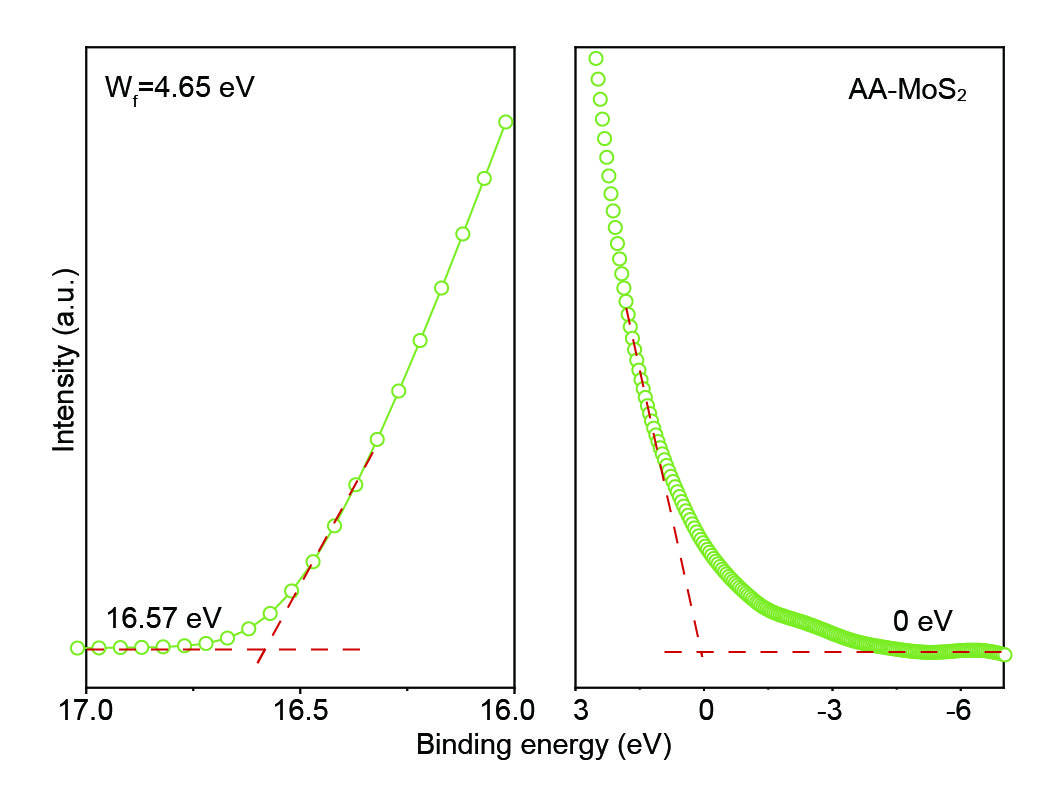


**Fig. S10.** UPS spectra of AA-MoS_2_.


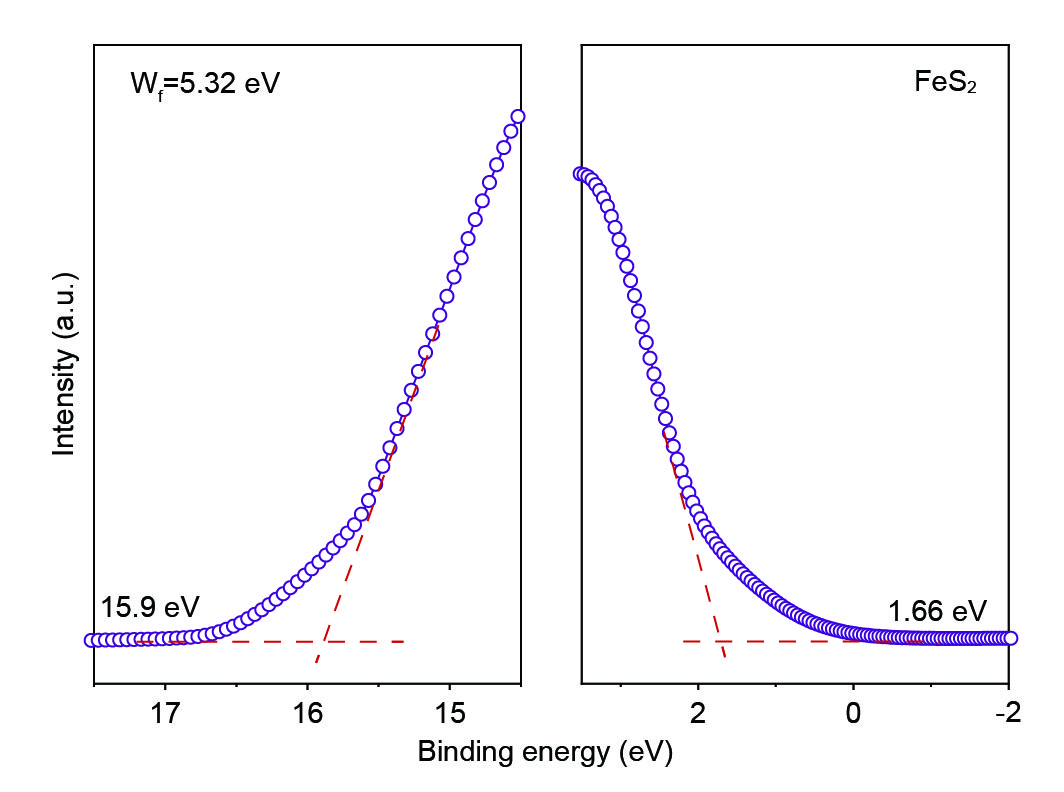


**Fig. S11.** UPS spectra of FeS_2_.


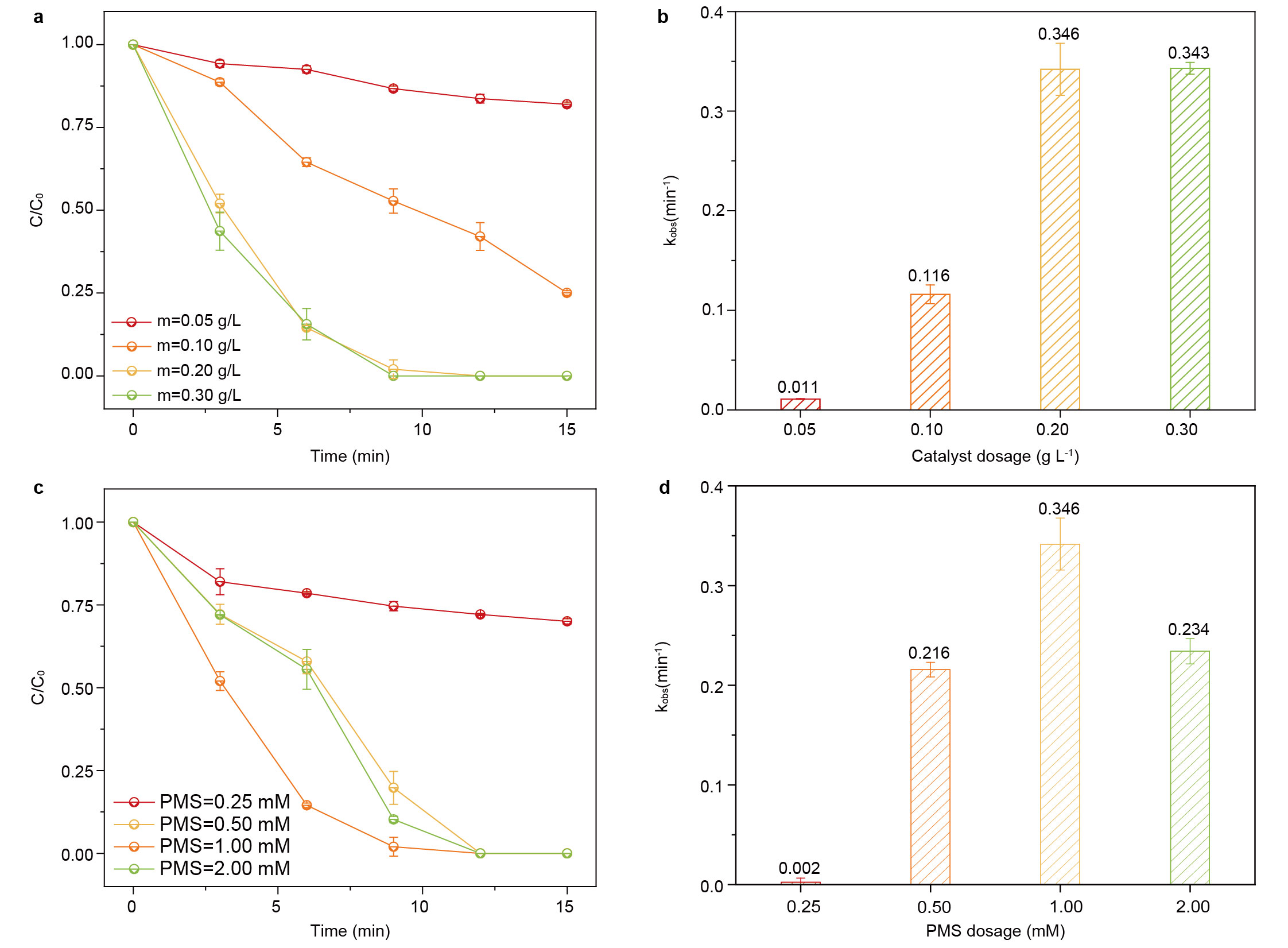


**Fig. S12. a-b**, The influence of PMS dosage on the APAP degradation in FeS_2_/MoS_2_/PMS systems; **c-d**, The influence of FeS_2_/MoS_2_ dosage on the APAP degradation in FeS_2_/MoS_2_/PMS systems.


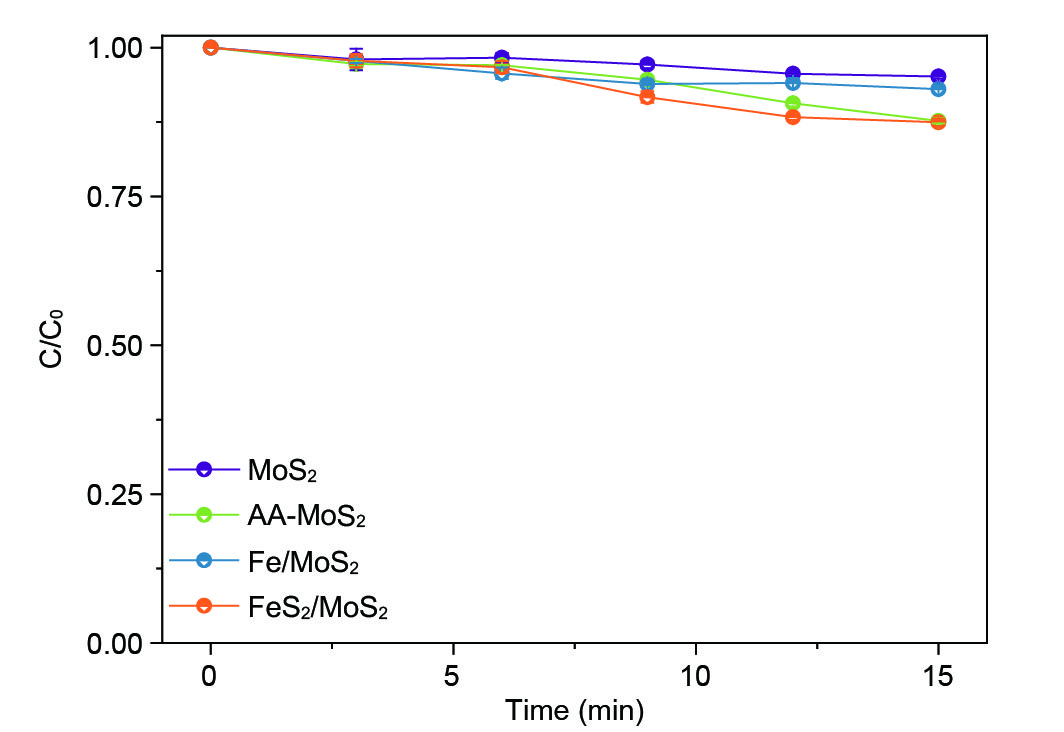


**Fig. S13.**  Adsorption behaviors of APAP in various systems.


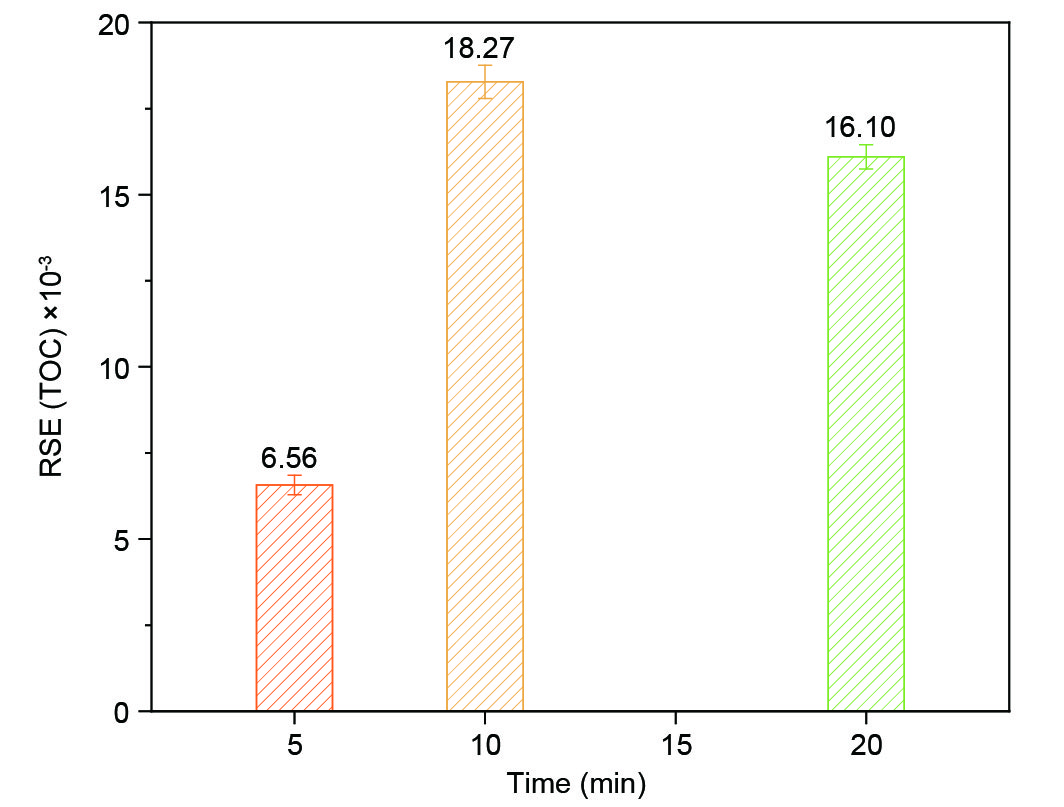


**Fig. S14.** Reaction stoichiometric efficiency (RSE) of FeS_2_/MoS_2_/PMS system.


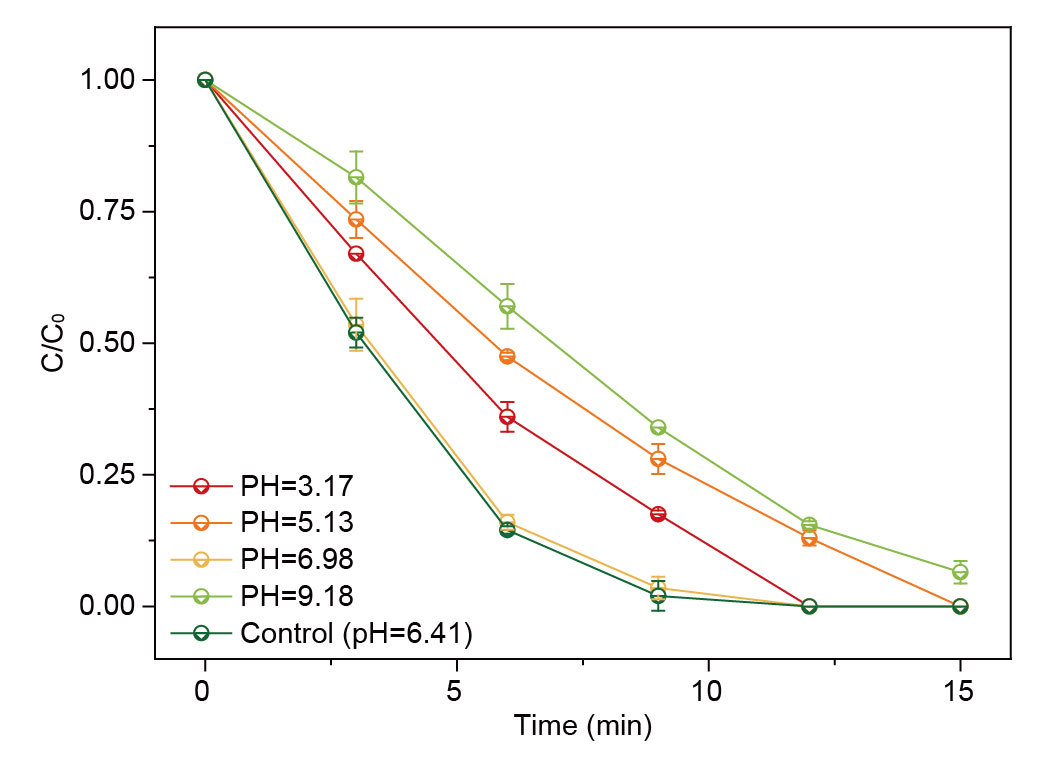


**Fig. S15.** The influence of pH on the APAP degradation in FeS_2_/MoS_2_/PMS systems.


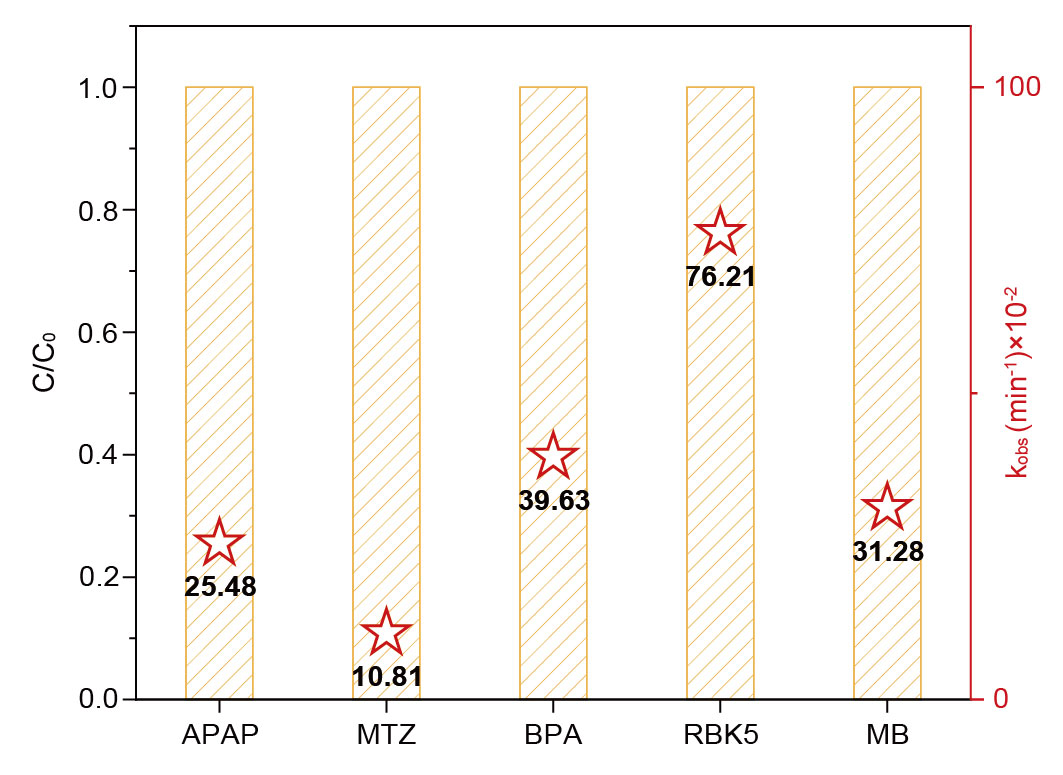


**Fig. S16.** Mixed pollutants degradation efficiency under FeS_2_/MoS_2_/PMS. (Reaction conditions: [Catalyst] = 1 g L^-1^, [APAP]_0_ = 10 mg L^-1^, [MTZ]_0_ = 10 mg L^-1^, [BPA]_0_ = 10 mg L^-1^, [RBK5]_0_ = 10 mg L^-1^, [MB]_0_ = 10 mg L^-1^, [PMS]_0_ = 5 mM, [Reaction time] = 15 min, T = 25 ℃).


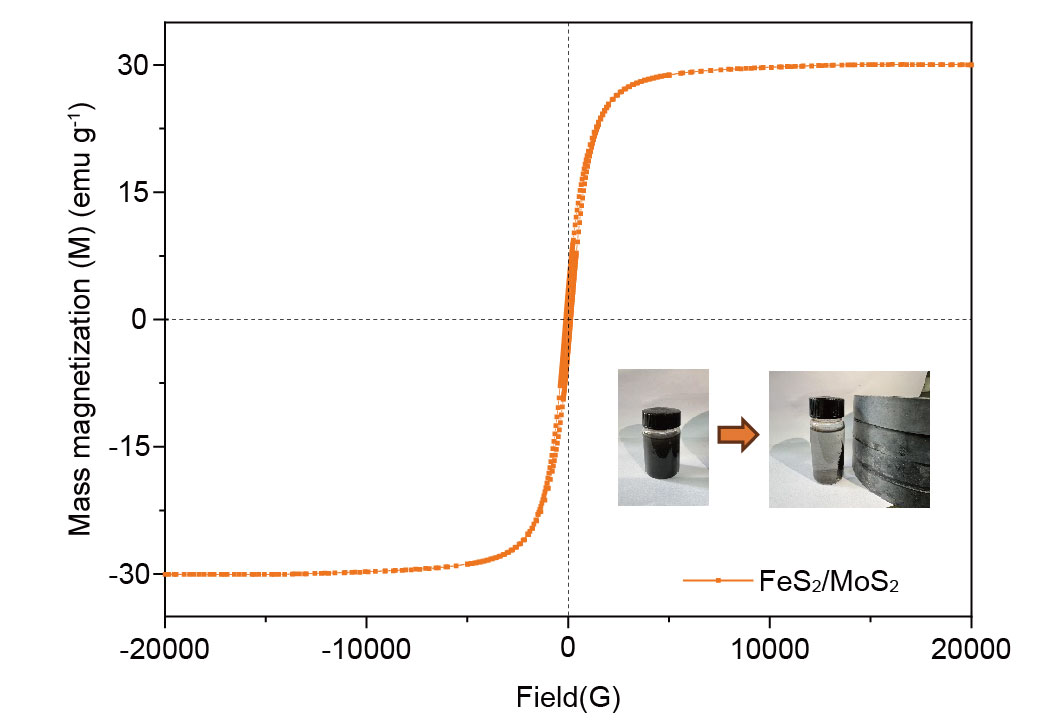


**Fig. S17.** VSM of FeS_2_/MoS_2_.


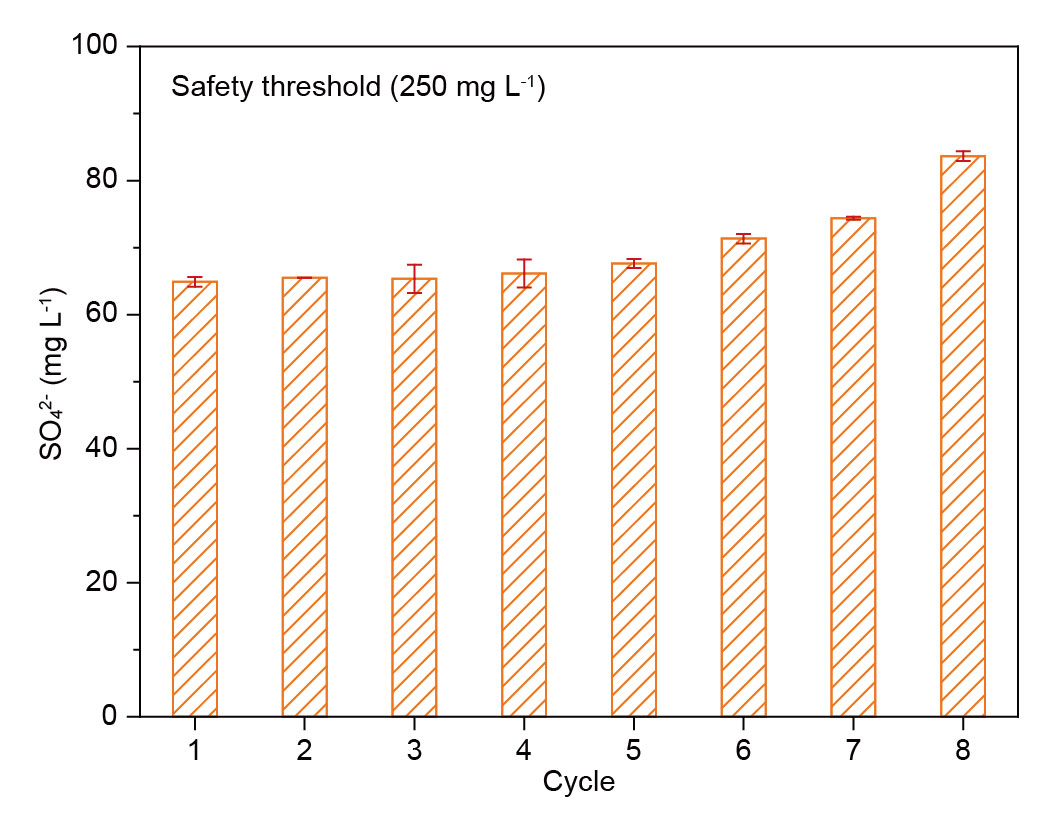


**Fig. S18.** SO_4_^2-^ concentration detected in eight consecutive catalytic cycles.


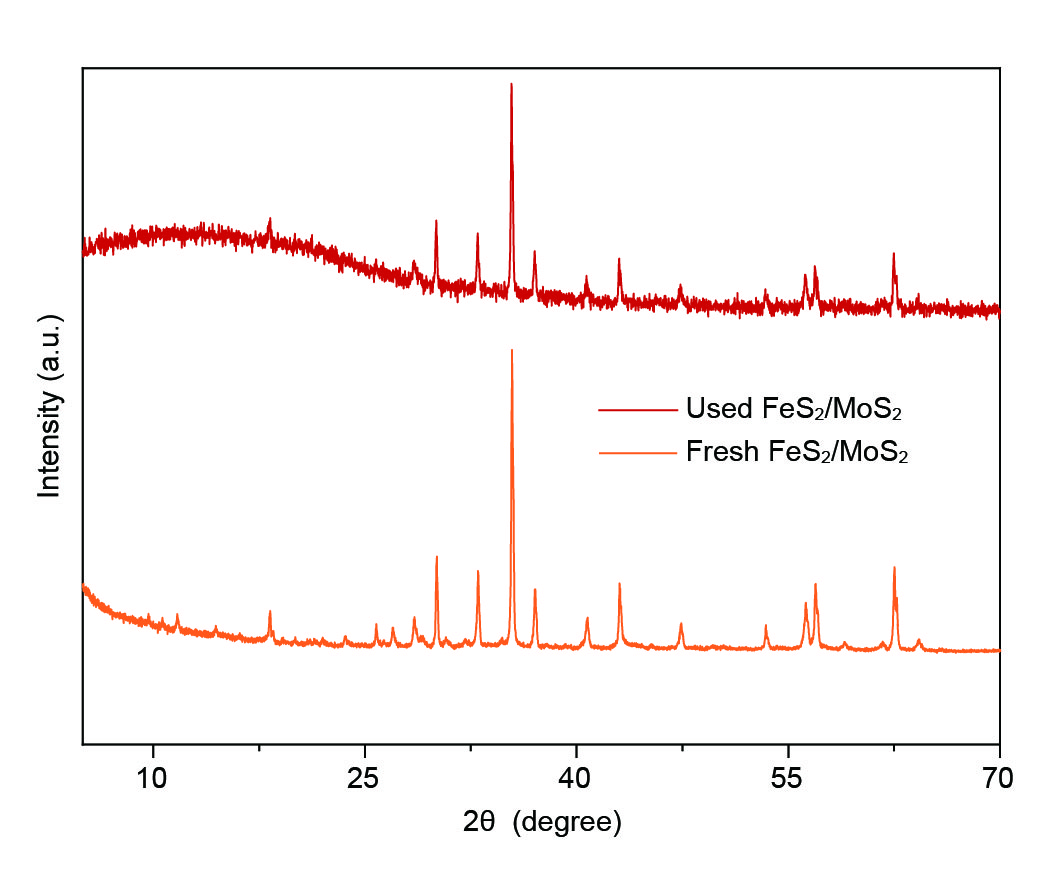


**Fig. S19.** XRD of the fresh and used FeS_2_/MoS_2_.

**
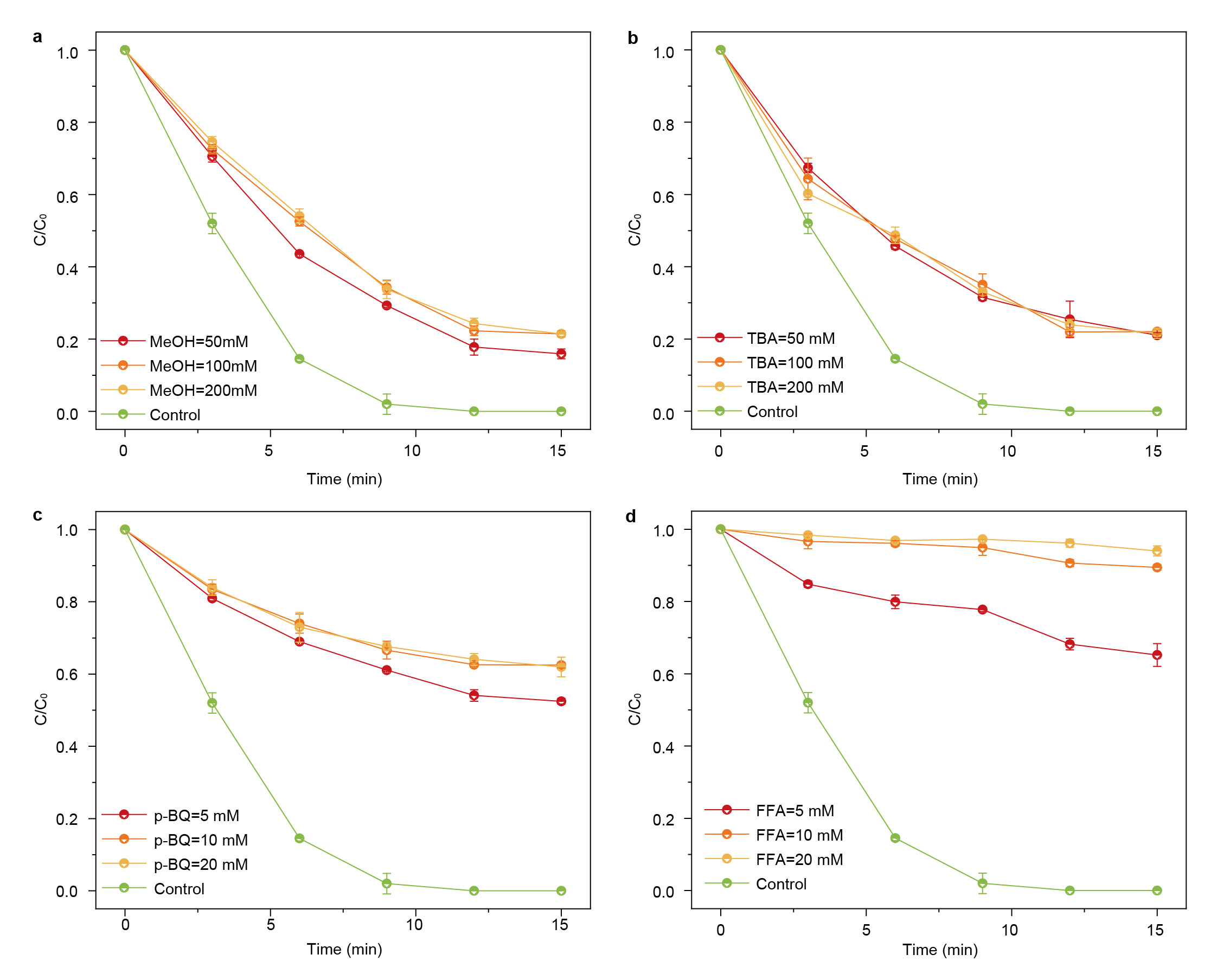
**

**Fig. S20.** Influence of different proportions of quencher on APAP degradation in FeS_2_/MoS_2_/PMS system. (PMS: Quencher = 1:5~500) (Reaction condition: [APAP] = 10 mg L^-1^, [PMS] = 1.0 mM, [catalyst] = 0.2 g L^-1^)


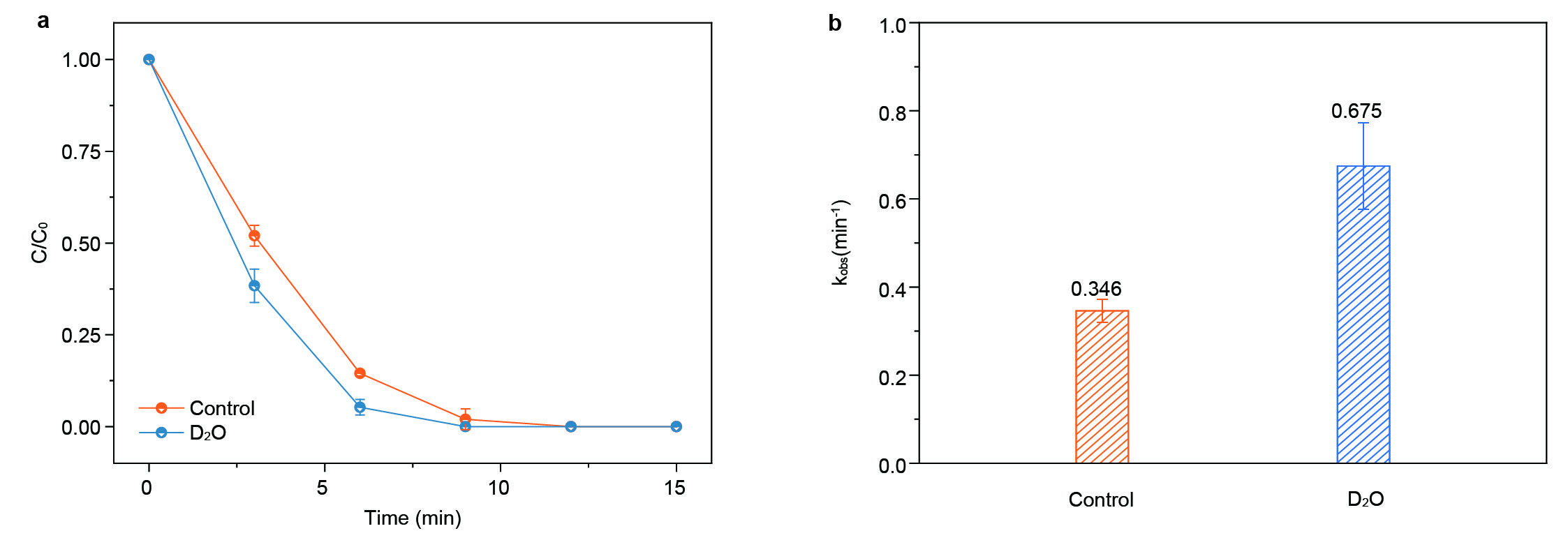


**Fig. S21. a-b,** APAP degradation (**a**) and reaction rate constants (**b**) in H_2_O and D_2_O within the FeS_2_/MoS_2_/PMS system. ([catalyst] = 0.2 g L^-1^, [PMS] = 1.0 mM, pH 7.0, [APAP] = 10 mg L^-1^.)


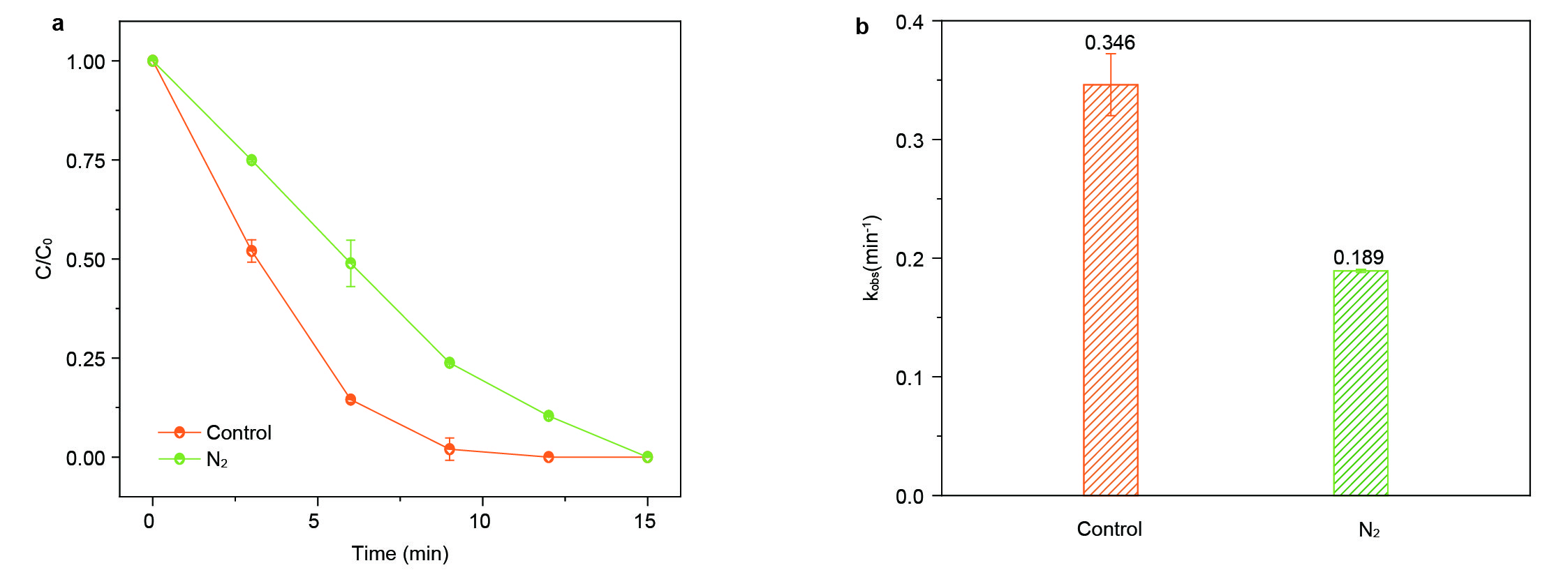


**Fig. S22.** APAP degradation rate constant in FeS_2_/MoS_2_/PMS systems with different atmospheres.


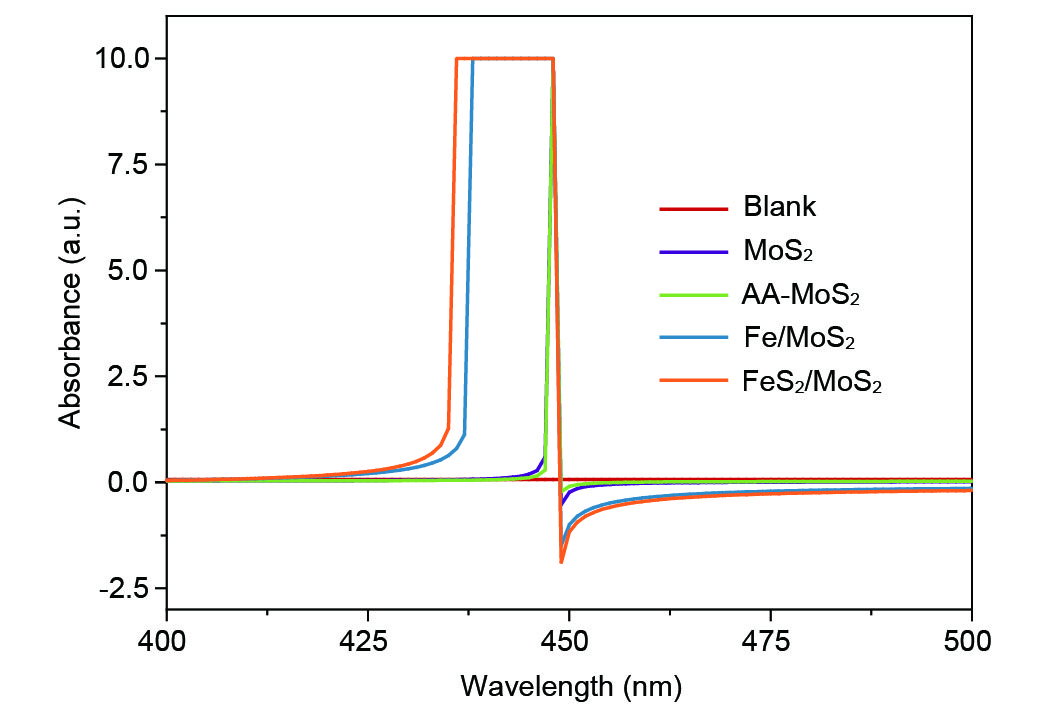


**Figure S23.** Absorption spectra of H_2_O_2_ under different catalytic systems obtained via titanium (IV) oxide bis(oxalate) potassium salt hydrate colorimetric reaction.


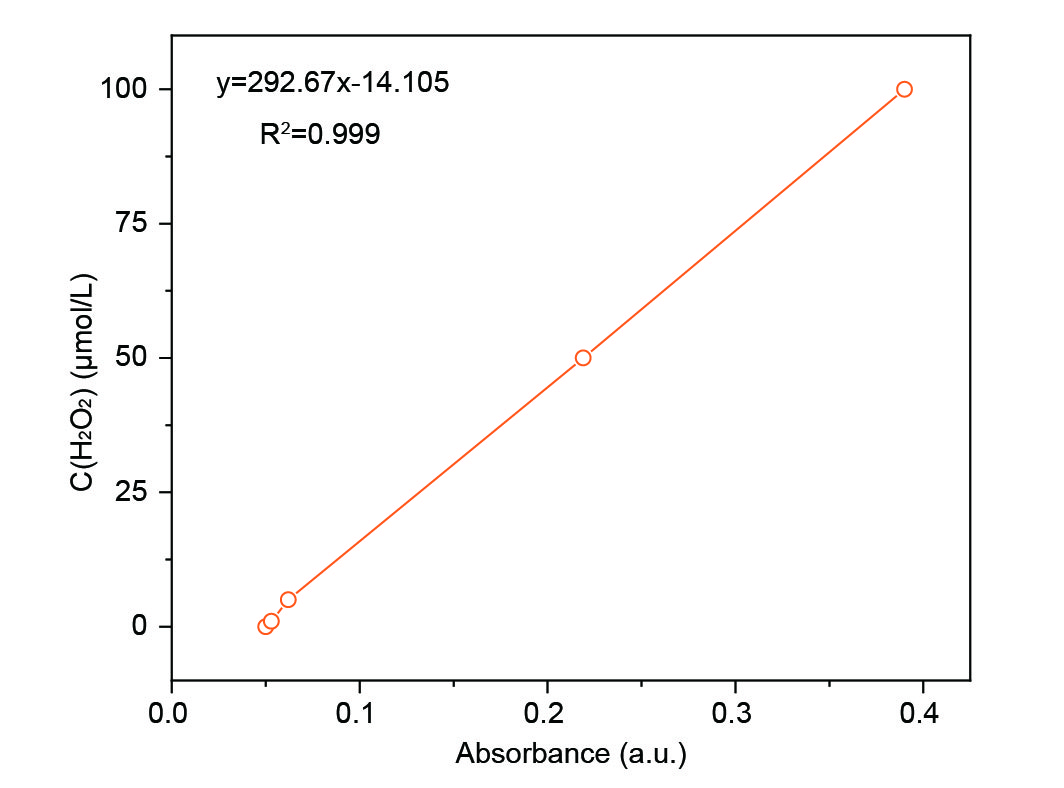


**Fig. S24.** H_2_O_2_ quantitative calibration curve.


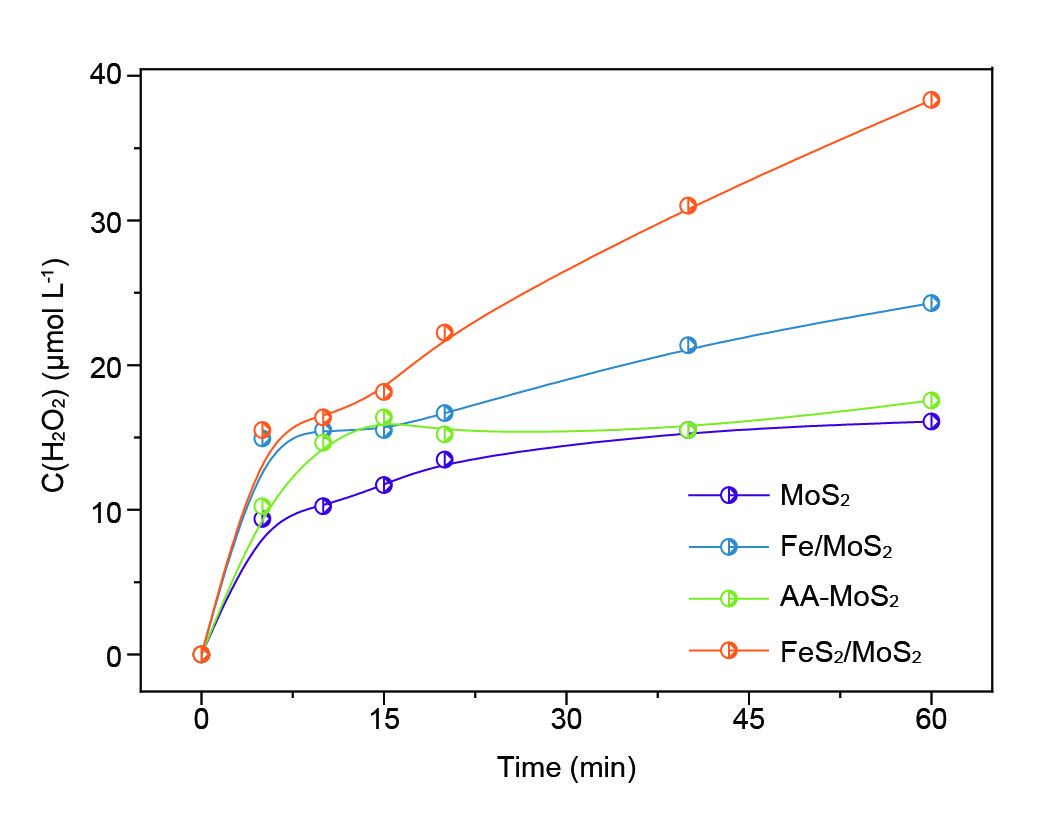


**Fig. S25.** H_2_O_2_ quantification of different catalysts.


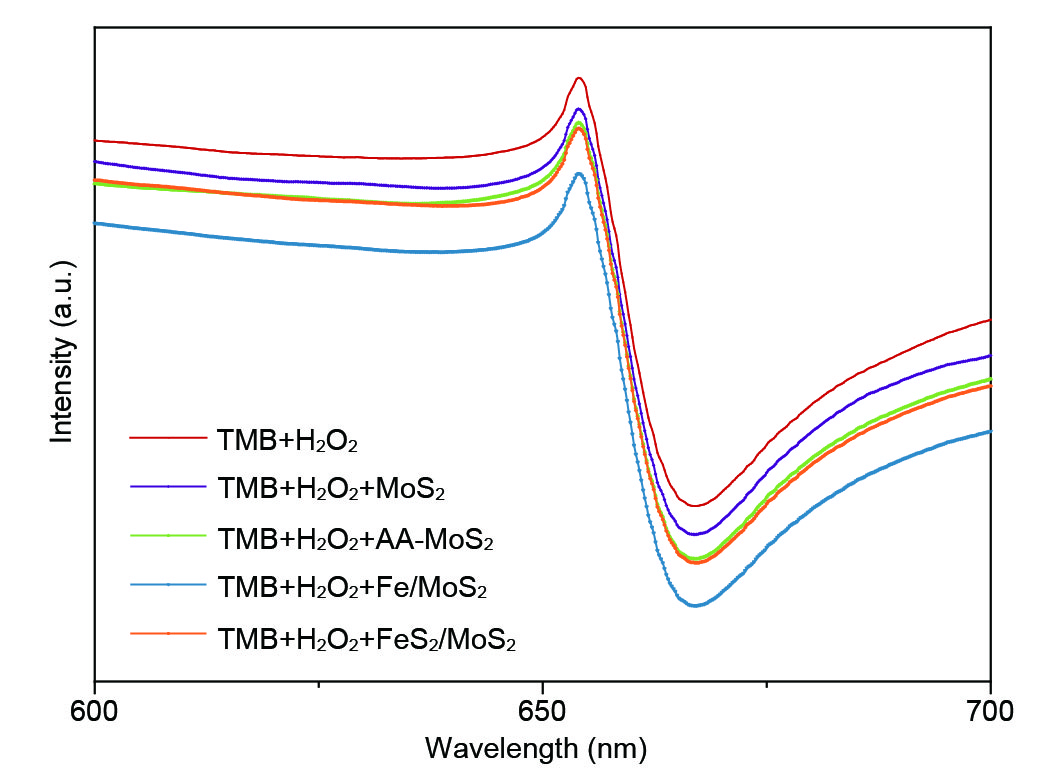


**Fig. S26.** The absorption spectra of TMB. (Condition: 8 μL of 20 mM TMB was added to 170 μL of acetate buffer (0.2 M, pH=3.6), and then 2 μL of 100 mM H_2_O_2_ and 20 μL of 1 mg L^-1^ catalysts).


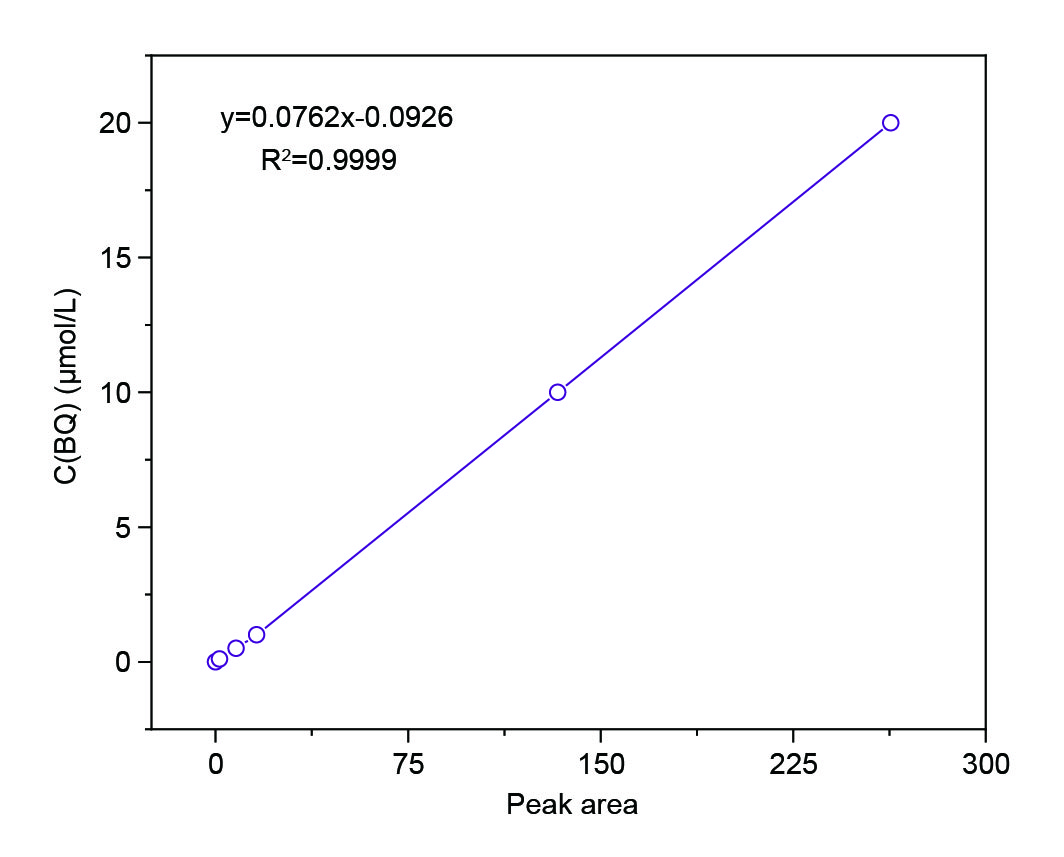


**Fig. S27.** BQ quantitative calibration curve.


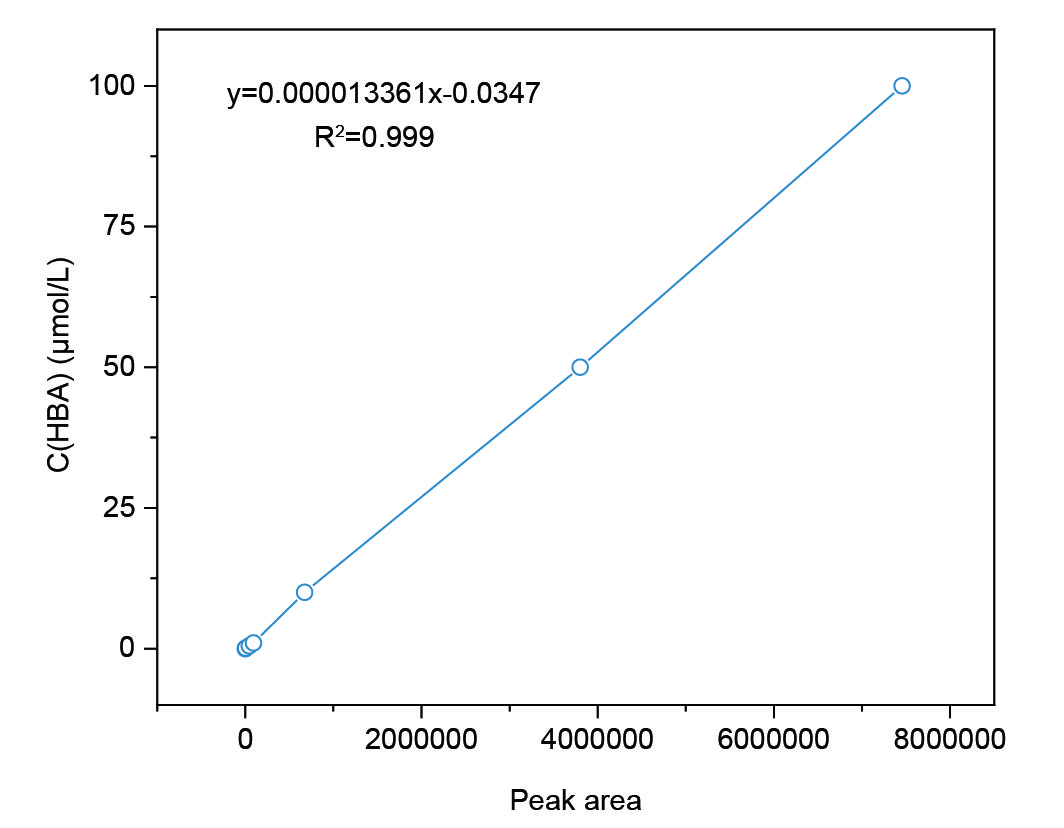


**Fig. S28.** HBA quantitative calibration curve.


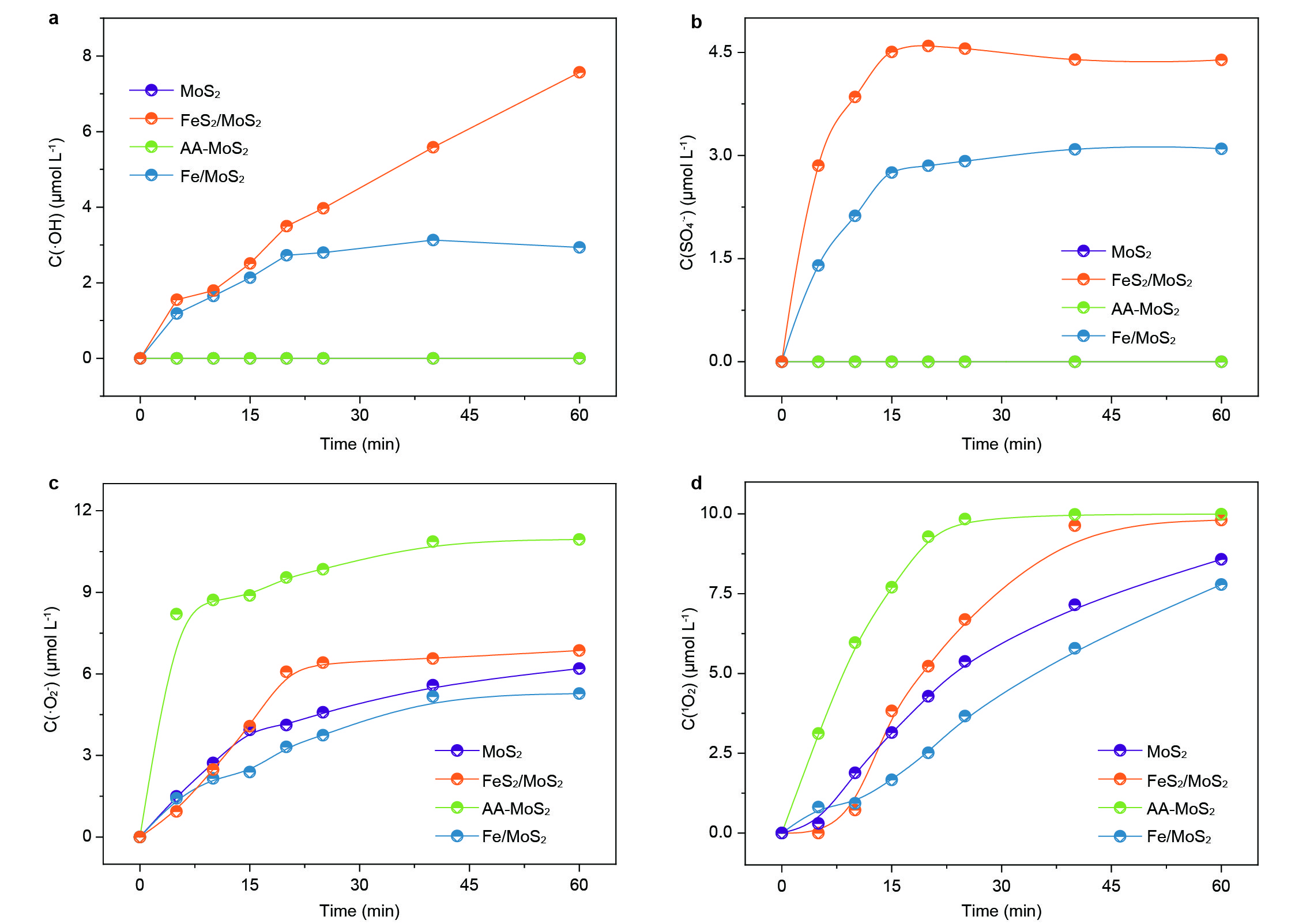


**Fig. S29.** **a-d**, ⋅OH (**a**), SO_4_^·-^ (**b**), ·O_2_^-^ (**c**), and ^1^O_2_ (**d**) quantification in different catalytic systems.


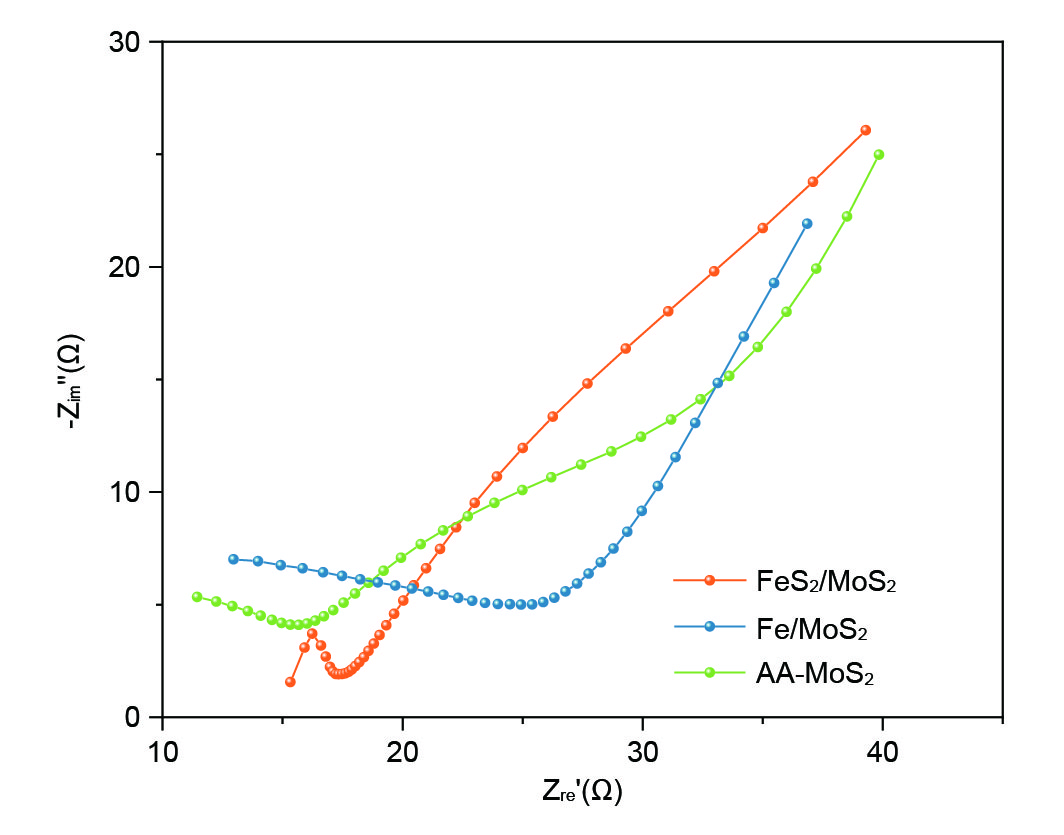


**Fig. S30.** Electrochemical impedance spectroscopy of different samples.


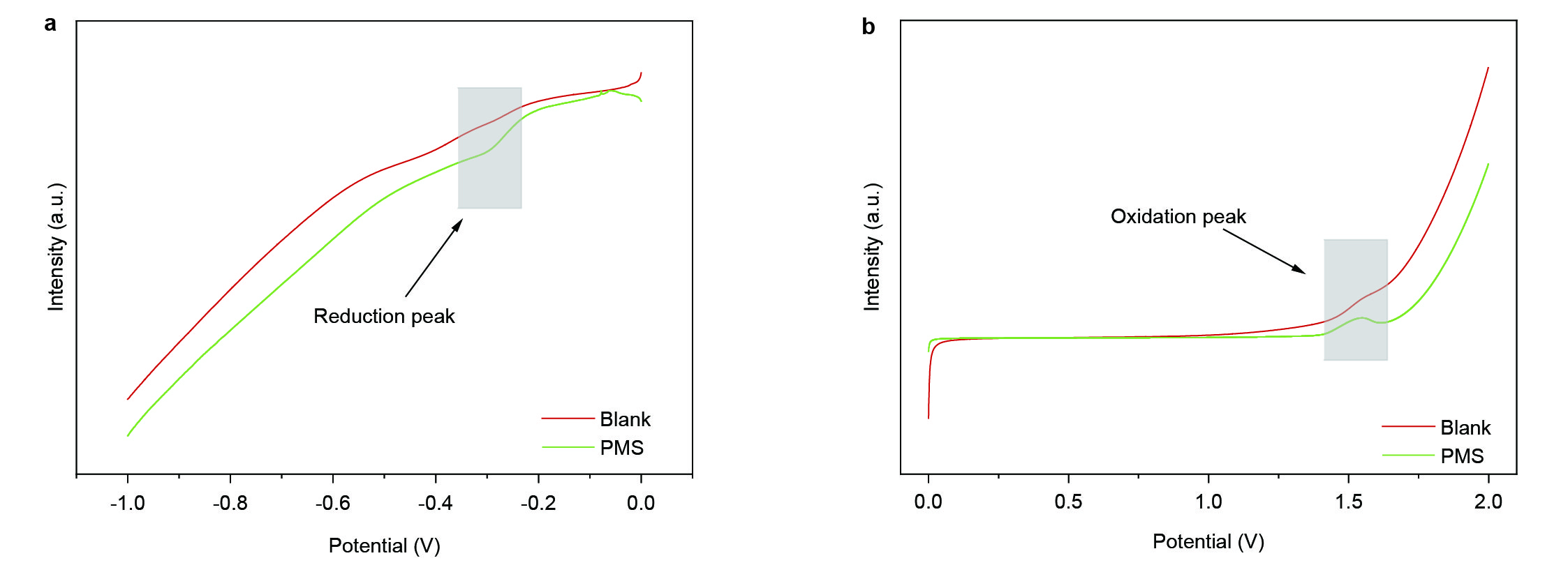


**Fig. S31.** Linear sweep voltammograms (LSV) curves of FeS_2_/MoS_2_ electrode captured with and without PMS: **a**, cathodic scan from 0 to -1.0 V; **b**, anodic scan from 0 to 2.0 V.


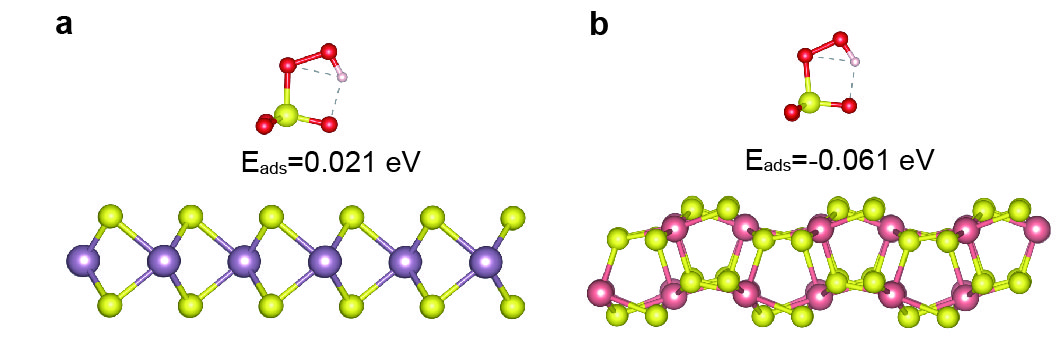


**Fig. S32.** **a-b**, Adsorption energy of PMS on MoS_2_ (**a**) and FeS_2_ (**b**).


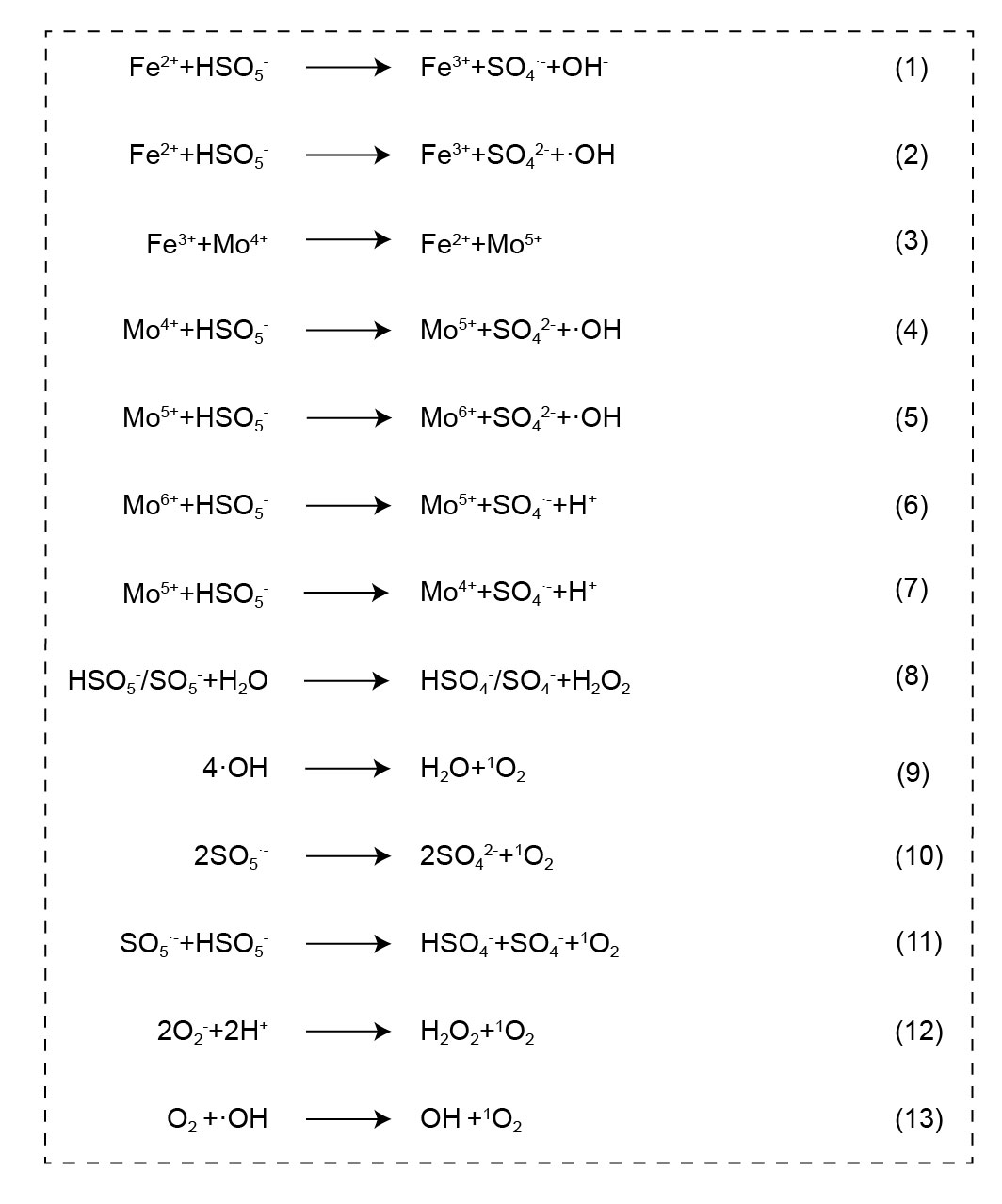


**Fig. S33.** ROS generation process in the FeS_2_/MoS_2_/PMS system.



















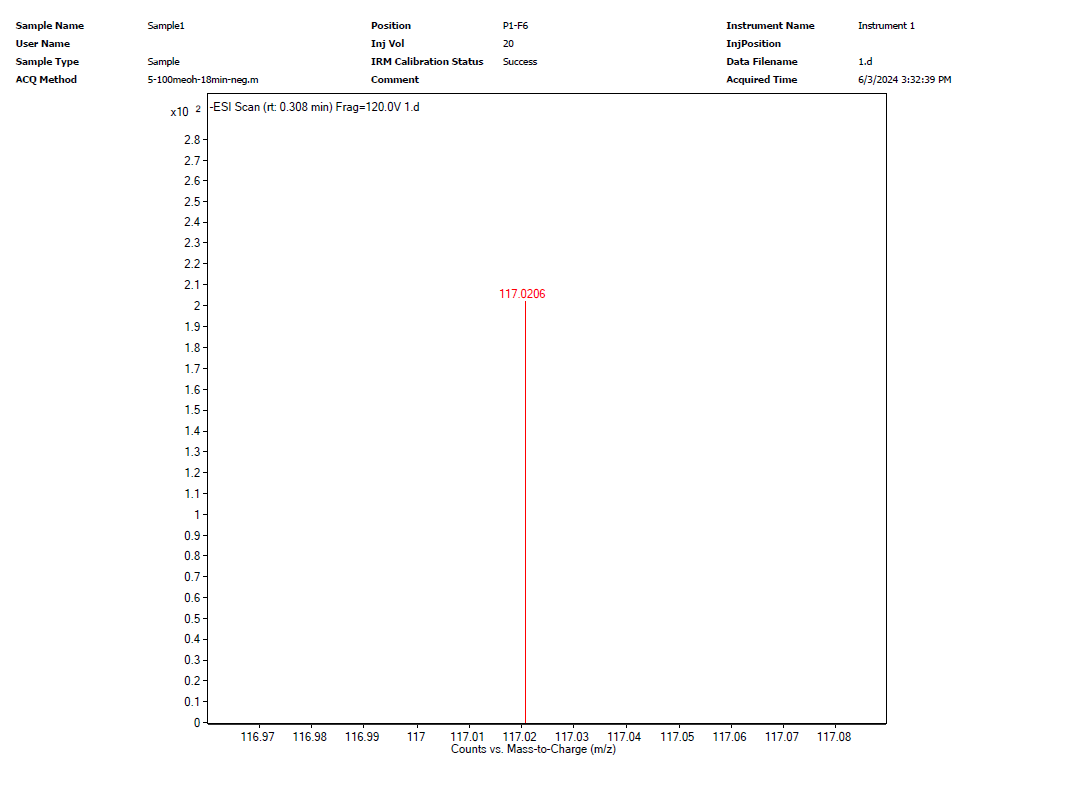











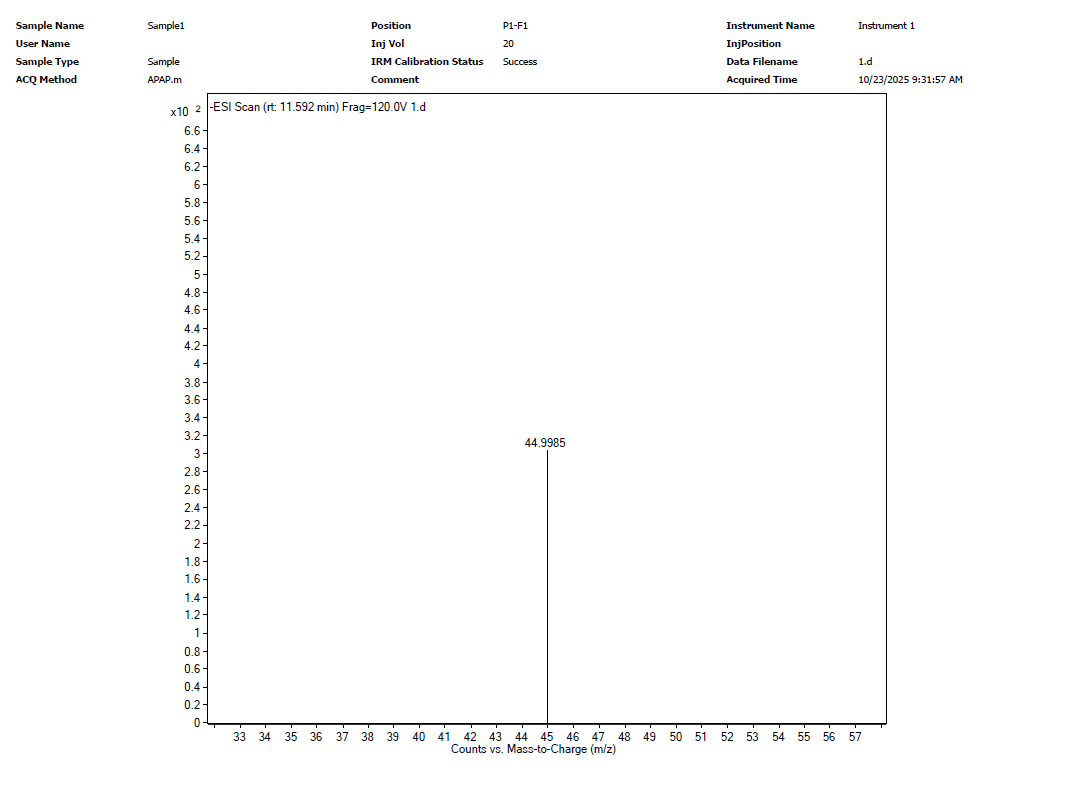


**Fig. S34.** Extraction of mass spectra of APAP degradation products in the FeS_2_/MoS_2_/PMS system.


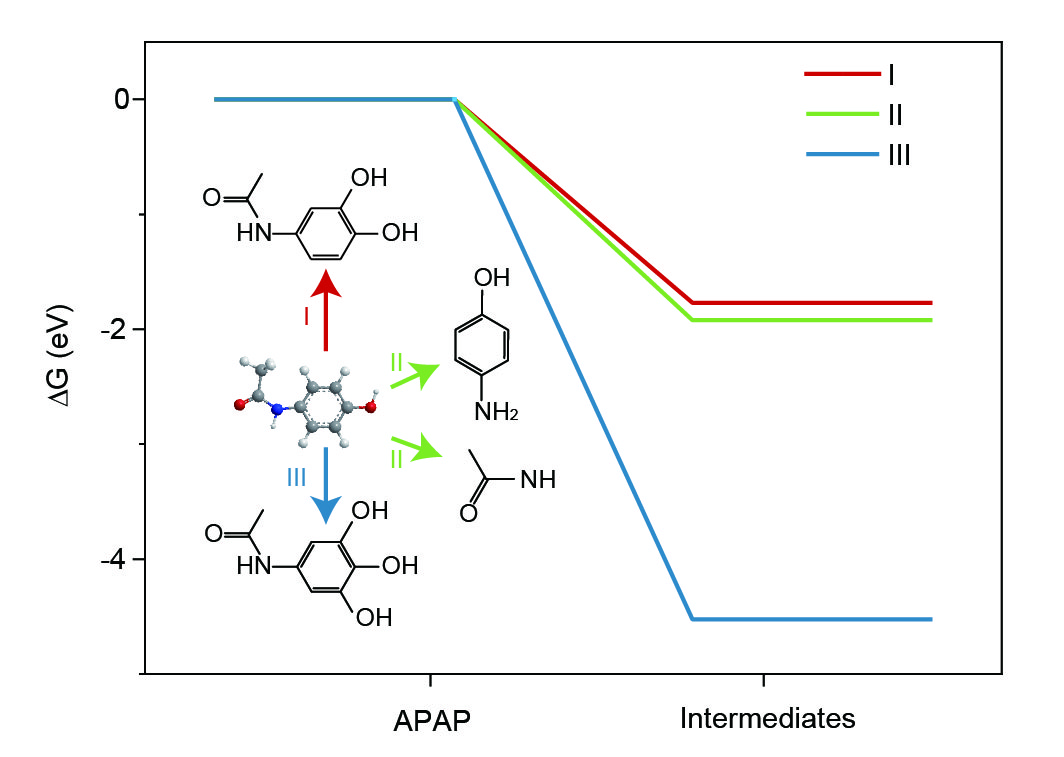


**Fig. S35.** Energy profile of the three reaction pathways for the initial APAP reaction.


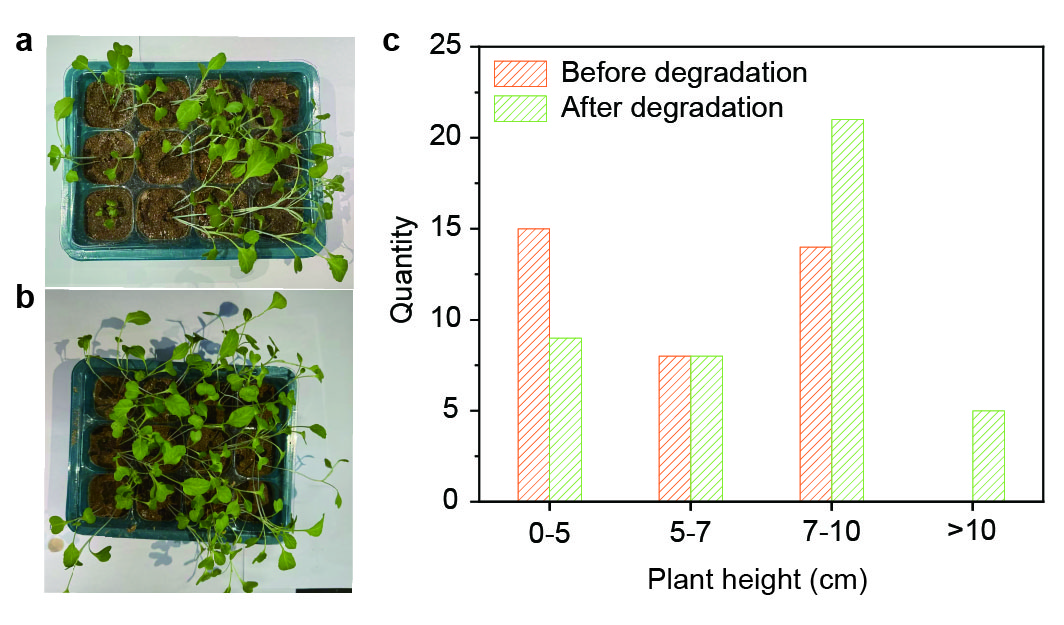


**Fig. S36. a-b,** Cauliflower cultivation in APAP-contaminated water (before degradation) (**a**) and treated water (after degradation) (**b**); **c**, Corresponding distribution of plant height.

**Table S1.** The HPLC analysis conditions for different substrates

| Substrates | Flow (mL/min) | λ (nm) | Mobile phase |
| --- | --- | --- | --- |
| Metronidazole (MTZ) | 1.0 | 318 | anhydrous/water = 20:80 |
| Naproxen (NPX) | 0.8 | 240 | methanol/0.01 mol/L potassium dihydrogen phosphate(pH=3) = 75:25 |
| Sulfamethoxazole (SMX) | 1.0 | 265 | 0.1% Acetic acid/ Acetonitrile = 60:40 |
| Carbamazepine (CBZ) | 1.0 | 280 | methanol/water = 60:40 |
| Norfloxacin (NOR) | 0.8 | 280 | acetonitrile/0.1% (v/v) formic acid = 15:85 |
| Bisphenol A (BPA) | 0.8 | 254 | methanol/water = 56:24 |
| Ciprofloxacin (CIP) | 1.0 | 278 | acetonitrile/0.3% formic acid = 25:75 |
| Acetaminophen (APAP) | 0.8 | 243 | methanol/water = 60:40 |
| Benzalkonium chloride (BAC) | 1.0 | 262 | 70 mmol/L [ammonium acetate](https://www.sciencedirect.com/topics/chemistry/ammonium-acetate) (pH=5) /water = 30:70 |
| Atrazine (ATZ) | 1.0 | 225 | methanol/water = 60/40 |

**Table S2.** Comparison of the catalytic performance of recently reported AOP reaction system for APAP degradation

| **Reaction system** | **Reaction condition** | | **pH** | **k (min^-1^)** | **Ref** |
| --- | --- | --- | --- | --- | --- |
| FeS_2_/MoS_2_ | | [APAP]=10.0 mg·L^-1^, [PMS]=1.0 mM,  [CAT]=0.20 g·L^-1^ | 3-9 | 0.346 | This work |
| Fe_3_O_4_ MNPs | | [APAP]=10.0 mg·L^-1^, [PMS]=0.2 mM,  [CAT]=0.20 g·L^-1^ | Neutral | 0.003 | [6] |
| Fe-ClBC | | [APAP]=10.0 mg·L^-1^,  [PMS]=0.3 mM,  [CAT]=0.10 g·L^-1^ | Neutral | 0.038 | [7] |
| FeOCl | | [APAP]=1.5 mg·L^-1^,  [PMS]=2.0 mM,  [CAT]=0.20 g·L^-1^ | 8.5 | 0.130 | [8] |
| TiO_2_/g-C_3_N_4_ (TCN) | | [APAP]=10 mg·L^-1^,  [PMS]=9.75 mM,  [CAT]=0.60 g·L^-1^ | Neutral | 0.024 | [9] |
| PB-Mn-800 | | [APAP]=10.0 mg·L^-1^,  [PMS]=1.0 mM,  [CAT]=0.10 g·L^-1^ | Neutral | 0.060 | [10] |
| NH_2_-BDC | | [APAP]=10.0 mg·L^-1^,  [PMS]=0.5 mM,  [CAT]=0.10 g·L^-1^ | 3-11 | 0.060 | [11] |
| 2sNBG800 | | [APAP]=10.0 mg·L^-1^,  [PMS]=0.5 mM,  [CAT]=0.20 g·L^-1^ | Neutral | 0.182 | [12] |
| A-FKCB | | [APAP]=10.0 mg·L^-1^,  [PMS]=0.5 mM,  [CAT]=0.10 g·L^-1^ | Neutral | 0.068 | [13] |

**Table S3.** Physicochemical information and classification of representative pollutants

| **No.** | **Pollutant name** | **Molecular formula** | **Chemical Structure** | **Category** |
| --- | --- | --- | --- | --- |
| 1 | Acetaminophen (APAP) | C_8_H_9_NO_2_ |  | PPCPs (Analgesic) |
| 2 | Naproxen (NPX) | C_14_H_14_O_3_ |  | PPCPs (NSAIDs) |
| 3 | Sulfamethoxazole (SMX) | C_10_H_11_N_3_O_3_S |  | PPCPs (Antibiotic) |
| 4 | Norfloxacin (NOR) | C_16_H_18_FN_3_O_3_ |  | PPCPs (Antibiotic) |
| 5 | Metronidazole (MTZ) | C_6_H_9_N_3_O_3_ |  | PPCPs (Antibiotic) |
| 6 | Ciprofloxacin (CIP) | C_17_H_18_FN_3_O_3_ |  | PPCPs (Antibiotic) |
| 7 | Carbamazepine (CBZ) | C_15_H_12_N_2_O |  | PPCPs (Anticonvulsant) |
| 8 | Tetracycline (TC) | C_22_H_24_N_2_O_8_ |  | PPCPs (Antibiotic) |
| 9 | Benzalkonium chloride (BAC) | C_21_H_38_ClN |  | PPCPs (Disinfectant/Surfactant) |
| 10 | Bisphenol A (BPA) | C_15_H_16_O_2_ |  | EDC  (Endocrine Disruptor) |

**Table S3** **(continued).** Physicochemical information and classification of representative pollutants

| **No.** | **Pollutant name** | **Molecular formula** | **Chemical Structure** | **Category** |
| --- | --- | --- | --- | --- |
| 11 | Atrazine (ATZ) | C_8_H_14_ClN_5_ |  | Pesticide (Herbicide) |
| 12 | Reactive black 5 (RBK5) | C_26_H_21_N_5_Na_4_O_19_S_6_ |  | Dye (Azo dye) |
| 13 | Methylene blue (MB) | C_16_H_18_ClN_3_S |  | Dye (Thiazine dye) |
| 14 | Crystal violet (CV) | C_25_H_30_ClN_3_ |  | Dye (Triphenylmethane) |
| 15 | Methyl orange (MO) | C_14_H_14_N_3_NaO_3_S |  | Dye (Azo dye) |

**Table S4.** Analysis of XPS results for catalyst

| XPS results | Mo 3d | | | Fe 2p | | | S 2p | | |
| --- | --- | --- | --- | --- | --- | --- | --- | --- | --- |
|  | Mo (IV) | Mo (V) | Mo (VI) | Fe (II) | Fe (III) | Fe (II)-S | S^0^ | S_n_^2-^ | S^2-^ |
| Fe/MoS_2_ | 88.2% |  | 11.8% | 65.1% | 34.9% |  | 43.2% | 25.0% | 31.8% |
| FeS_2_/MoS_2_ | 82.3% | 7.7% | 10.1% | 59.0% | 24.1% | 16.9% | 23.1% | 17.8% | 59.0% |
| Used  FeS_2_/MoS_2_ | 67.3% | 22.6% | 10.1% | 60.8% | 25.6% | 13.6% | 14.0% | 20.6% | 59.4% |

**Table S5.** Fukui function values of APAP

| Atom index | OW f^+^ | OW f^-^ | OW f^0^ |
| --- | --- | --- | --- |
| 1(C) | 0.08067 | 0.09917 | 0.08992 |
| 2(C) | 0.1145 | 0.08319 | 0.09885 |
| 3(C) | 0.10203 | 0.08278 | 0.09241 |
| 4(C) | 0.0836 | 0.09534 | 0.08947 |
| 5(C) | 0.09827 | 0.08722 | 0.09274 |
| 6(C) | 0.10488 | 0.08471 | 0.0948 |
| 7(H) | 0.02119 | 0.00612 | 0.01366 |
| 8(H) | 0.01934 | 0.00623 | 0.01279 |
| 9(H) | 0.01247 | 0.00657 | 0.00952 |
| 10(H) | 0.01219 | 0.00944 | 0.01081 |
| 11(O) | 0.03276 | 0.08521 | 0.05898 |
| 12(H) | 0.02861 | 0.00546 | 0.01703 |
| 13(N) | 0.04401 | 0.09569 | 0.06985 |
| 14(H) | 0.0254 | 0.00757 | 0.01648 |
| 15(C) | 0.02493 | 0.01774 | 0.02133 |
| 16(H) | 0.0129 | 0.00209 | 0.0075 |
| 17(H) | 0.01067 | 0.00345 | 0.00706 |
| 18(H) | 0.01269 | 0.00208 | 0.00738 |
| 19(C) | 0.09443 | 0.0392 | 0.06682 |
| 20(O) | 0.06443 | 0.18074 | 0.12259 |

**Table S6.** Initial Reaction Pathway Energy Data.

| **No.** | **Pollutant name** | **Molecular formula** | **Chemical Structure** | **Energy (eV)** |
| --- | --- | --- | --- | --- |
| 1 | Acetaminophen (APAP) | C_8_H_9_NO_2_ |  | -14026.35 |
| 2 | P1 | C_8_H_9_NO_3_ |  | -16072.37 |
| 3 | P5 | C_6_H_7_NO |  | -5692.63 |
| 4 | P7 | C_2_H_5_NO |  | -9872.17 |
| 5 | P9 | C_8_H_9_NO_4_ |  | -18119.07 |
| 6 | Hydrogen molecule | H_2_ |  | -15.65 |
| 7 | Nitrogen molecule | N_2_ |  | -1489.75 |
| 8 | Oxygen molecule | O_2_ |  | -2044.10 |

**Table S7.** Estimated acute and chronic toxicity for fish, daphnid and green algae of APAP and transformation products by ECOSAR.

| Compound | Acute toxicity ^a^ | | | Chronic toxicity ^a^ | | |
| --- | --- | --- | --- | --- | --- | --- |
|  | Fish  (96-h LC_50_) | Daphnid  (48-h LC_50_) | Green algae  (96-h EC_50_) | Fish  (ChV) | Daphnid  (ChV) | Green algae  (ChV) |
| APAP | 15.5 | 0.874 | 2.22 | 0.124 | 0.189 | 0.352 |
| P1 | 246 | 5030 | 22.9 | 195 | 2180 | 2.15 |
| P2 | 501 | 12500 | 34.5 | 430 | 5600 | 3 |
| P3 | 144 | 2740 | 14.9 | 111 | 1170 | 1.44 |
| P4 | 164000 | 74100 | 21300 | 12200 | 3810 | 3350 |
| P5 | 240 | 47 | 283 | 19.8 | 3.78 | 27.6 |
| P6 | 22.2 | 256 | 4.9 | 14.2 | 10.1 | 0.568 |
| P7 | 176 | 3430 | 17.6 | 137 | 1470 | 1.68 |
| P8 | 6130 | 2770 | 807 | 458 | 144 | 127 |
| P9 | 216 | 145 | 456 | 459 | 11 | 14.9 |
| P10 | 8.89 | 8.33 | 4.75 | 1.45 | 0.0885 | 1.79 |
| P11 | 418 | 224 | 130 | 38.1 | 18.5 | 29.9 |

^a^ Unit = mg/ L.

LC_50_/EC_50_/ChV ≤ 1 **Very toxic**

1 < LC_50_/EC_50_/ChV ≤ 10 **Toxic**

10 < LC_50_/EC_50_/ChV ≤ 100 **Harmful**

LC_50_/EC_50_/ChV > 100 **Not harmful**

**References:**

[1] J. Hutter, M. Iannuzzi, F. Schiffmann, J. VandeVondele, cp2k: atomistic simulations of condensed matter systems, WIREs Comput. Mol. Sci. 4 (2014) 15-25. https://doi.org/10.1002/wcms.1159.

[2] J. VandeVondele, M. Krack, F. Mohamed, M. Parrinello, T. Chassaing, J. Hutter, Quickstep: Fast and accurate density functional calculations using a mixed Gaussian and plane waves approach, Comput. Phys. Commun. 167 (2005) 103-128. https://doi.org/10.1016/j.cpc.2004.12.014.

[3] J.P. Perdew, K. Burke, M. Ernzerhof, Generalized Gradient Approximation Made Simple, Phys Rev Lett 77 (1996) 3865-3868. https://doi.org/10.1103/PhysRevLett.77.3865.

[4] S. Goedecker, M. Teter, J. Hutter, Separable dual-space Gaussian pseudopotentials, Phys Rev B 54 (1996) 1703-1710. https://doi.org/10.1103/PhysRevB.54.1703.

[5] S. Grimme, J. Antony, S. Ehrlich, H. Krieg, A consistent and accurate ab initio parametrization of density functional dispersion correction (DFT-D) for the 94 elements H-Pu, J. Chem. Phys. 132 (2010) 154104. https://doi.org/10.1063/1.3382344.

[6] C. Tan, N. Gao, Y. Deng, J. Deng, S. Zhou, J. Li, X. Xin, Radical induced degradation of acetaminophen with Fe_3_O_4_ magnetic nanoparticles as heterogeneous activator of peroxymonosulfate, J. Hazard. Mater. 276 (2014) 452-460. https://doi.org/10.1016/j.jhazmat.2014.05.068.

[7] Y. Zeng, H. Luo, D. He, J. Li, A. Zhang, J. Sun, J. Xu, X. Pan, Influence mechanism of anions on iron doping into swine bone char: Promoting non-radical oxidation of acetaminophen in a Fenton-like system, Sci. Total Environ. 920 (2024) 170982. https://doi.org/10.1016/j.scitotenv.2024.170982.

[8] C. Tan, Q. Xu, T. Sheng, X. Cui, Z. Wu, H. Gao, H. Li, Reactive oxygen species generation in FeOCl nanosheets activated peroxymonosulfate system: Radicals and non-radical pathways, J. Hazard. Mater. 398 (2020) 123084. https://doi.org/10.1016/j.jhazmat.2020.123084.

[9] H. Jiang, J. Yang, X. Wang, A. Wang, Y. li, J. Wang, Efficient Activation of Persulfate by TiO_2_/g-C_3_N_4_ Composite for Degradation of Acetaminophen Under Visible Light, Water. Air. Soil Pollut. 234 (2023) 619. https://doi.org/10.1007/s11270-023-06638-5.

[10] Z. Zhu, Y. Xue, T. Zhang, M. Xu, Q. Zhang, J. Hong, Applying the carbon vacancy-enhanced catalyst as the surface reactor for peroxymonosulfate activation and efficient Acetaminophen degradation: Performance and mechanism, Sep. Purif. Technol. 326 (2023) 124811. https://doi.org/10.1016/j.seppur.2023.124811.

[11] Y. Xue, R. Gao, S. Lin, Q. Zhong, Q. Zhang, J. Hong, Regulating the interface electron distribution of iron-based MOFs through ligand functionalization enables efficient peroxymonosulfate utilization and catalytic performance, J. Colloid Interface Sci. 663 (2024) 358-368. https://doi.org/10.1016/j.jcis.2024.02.118.

[12] X. Chen, X. Duan, W.-D. Oh, P.-H. Zhang, C.-T. Guan, Y.-A. Zhu, T.-T. Lim, Insights into nitrogen and boron-co-doped graphene toward high-performance peroxymonosulfate activation: Maneuverable N-B bonding configurations and oxidation pathways, Appl. Catal. B Environ. 253 (2019) 419-432. https://doi.org/10.1016/j.apcatb.2019.04.018.

[13] X. Sun, K. Wu, G. Yang, F. Shen, S. Zhang, T. Wu, S. Sun, Y. Jiang, X. Wu, Synergistic effect of carbon defects and iron active sites in biochar-activated peroxymonosulfate for acetaminophen degradation: Unlocking the non-radical mechanisms, Chem. Eng. J. 523 (2025) 168775. https://doi.org/10.1016/j.cej.2025.168775.
